# Nanogel Dressing with Targeted Glucose Reduction and pH/Hyaluronidase Dual-Responsive Release for Synergetic Therapy of Diabetic Bacterial Wounds

**DOI:** 10.3390/gels11060380

**Published:** 2025-05-22

**Authors:** Wanhe Luo, Yongtao Jiang, Jinhuan Liu, Samah Attia Algharib, Ali Sobhy Dawood, Shuyu Xie

**Affiliations:** 1 Engineering Laboratory for Tarim Animal Diseases Diagnosis and Control, College of Animal Science and Technology, Tarim University, Alar 843300, China; jiangyongtao2022@163.com (Y.J.); liujinhuan0830@163.com (J.L.); 2 College of Veterinary Medicine, Sichuan Agricultural University, Chengdu 611130, China; 3 Department of Clinical Pathology, Faculty of Veterinary Medicine, Benha University, Moshtohor 13736, Egypt; samah.alghareeb@fvtm.bu.edu.eg; 4 Infectious Diseases, Faculty of Veterinary Medicine, University of Sadat City, Sadat City 32897, Egypt; ali.dawood@vet.usc.edu.eg; 5 National Reference Laboratory of Veterinary Drug Residues (HZAU), Huazhong Agricultural University, Wuhan 430070, China

**Keywords:** nanogel dressing, pH/hyaluronidase dual responsiveness, glucose oxidase, diabetic bacteria wounds, synergetic therapy

## Abstract

The hyperglycemic microenvironment in diabetic wounds predisposes them to bacterial infections, sustains chronic inflammation, and hinders therapeutic efficacy. In this study, antibiotic-loaded fast-crosslinked hybrid nanogel wound dressings (florfenicol nanogels) based on Schiff’s base bond were obtained through N, O-carboxymethyl chitosan (N, O-CMCS) and oxidized hyaluronic acid (OHA). The successfully prepared florfenicol N, O-CMCS/OHA nanogels exhibited obvious pH- and HAase-responsiveness release, which allowed it to quickly release florfenicol at infected wounds to exert on-demand antibacterial activity, as well as accelerate diabetic bacterial-infected wound healing. The nanogel dressings showed excellent antibacterial activity by destroying the bacterial cell membrane and wall. More specifically, the glucose oxidase in the dressings can catalyze the breakdown of high-concentration glucose, generating abundant ROS that directly cause cellular damage. According to the results of wound healing, the dressings showed satisfactory anti-inflammatory and therapeutic effects for the full-thickness mouse skin defect wounds. The nanogel dressings are anticipated to be excellent wound dressings to synergistically overcome the theraputic difficulty of diabetic bacterial wounds.

## 1. Introduction

Diabetes is a long-term chronic condition brought on by either inadequate pancreatic synthesis of insulin or inappropriate use of the stored insulin [1]. It has a high mortality rate and can cause severe complications, including neuropathy, microangiopathy, and diabetic wounds, which often lead to amputation or death—especially in advanced cases of diabetic wounds [2,3]. At present, there is no effective method to completely treat diabetic wounds due to their long healing process, the persistently high blood sugar level, and related complications [4]. Thus, the thorough treatment of diabetic wounds is an urgent problem to be solved. Worryingly, the high blood sugar in the wound provides an ideal living environment for bacterial growth, leading to most diabetic wounds being accompanied by bacterial infections, including *Staphylococcus aureus* (*S. aureus*) and *Escherichia coli* (*E. coli*) [5,6,7]. The initial manifestation of diabetic wound infection may be the colonization of pathogenic bacteria in the wound, while the high blood sugar level of diabetic wounds is very conducive to the growth and reproduction of bacteria [8]. Research has shown that diabetes wounds containing bacteria will exacerbate wound infection, prolong inflammation, and delay wound healing time [9,10]. More importantly, bacterial persistence on the surface of the diabetic wounds further increases bacterial resistance, making it impossible to proceed with further treatment [11]. Therefore, it is a critical issue to reduce bacterial infection for diabetic wound therapy. The most common types of wound dressings are classic ones like bandages, gauzes, and disinfecting cottons due to their low cost and simple manufacturing process [12,13,14]. However, some drawbacks limit their application, such as providing limited protection and infection prevention, that the wound cannot maintain a moist environment, and that they are prone to adhere to the wound tissue. Additionally, changing the dressing can easily tear the wound; the healing effect of diabetic wounds can be unsatisfactory; and they can fail to eradicate bacteria in diabetic wounds [4,15,16]. On the other hand, hydrogels as modern wound dressings can retain wounds moisture, have better biocompatibility and degradability, and can greatly promote wound healing. Hydrogel wound dressings are mainly formed by single or mixed hydrated polymers through covalent or non-covalent crosslinking to achieve high moisture absorption and maintain a moist wound environment [17]. More importantly, hydrogel wound dressings can be replaced or removed without seriously damaging the wound tissue compared to traditional wound dressings [18]. In addition, hydrogel wound dressings are flexible and can be applied to the wound without squeezing the wound, and can absorb exudate, penetrate oxygen and metabolites, and hinder the entry of bacteria, thereby reducing the number of bacteria. Thus, with increasing bacterial resistance, developing safe and targetable antibiotic-loaded hydrogel wound dressings to enhance antibacterial activity and accelerate the healing of diabetes wounds containing bacteria has become an effective strategy [19]. Antibiotic-loaded hydrogel wound dressings can target the release of antibiotics at the site of bacterial infection and the occurrence of bacterial resistance by enhancing the antibacterial activity of their payload antibiotics, thereby accelerating the healing of diabetic wounds [17]. For example, quercetin and oleic acid hydrogels have been shown to drastically shorten the required time for wound healing, enhance tissue viscoelasticity, and lessen discomfort in diabetic ulcer patients [20]. In addition to having positive benefits on diabetic ulcers, the in vitro proliferation, migration, and angiogenesis of human umbilical vein endothelial cells are also positively impacted by the sodium alginate composite hydrogels. Moreover, the hydrogels increase the expression of important genes responsible for the healing of bacterial diabetic wounds, such as vascular endothelial growth factor (VEGF) and hypoxia-inducible factor-1α (HIF-1α) [21]. Furthermore, the excipients of hydrogel wound dressings are crucial. Chitosan (CS) is a natural polymer with excellent biodegradability, biocompatibility, and hemostatic and antibacterial properties [22]. The drawback is that CS is difficult to dissolve in water, which limits its application. But interestingly, CS can be carboxylated with monochloroacetic acid (MCAA) to obtain N, O-carboxymethyl chitosan (N, O-CMCS) with amino groups (-NH_2_) and good solubility. N, O-CMCS has a certain inhibitory effect against a broad range of Gram-positive bacteria such as *S. aureus,* and Gram-negative bacteria such as *E. coli*. Moreover, after binding with water molecules, N, O-CMCS can affect cell metabolism and growth through various pathways, thereby moisturizing and protecting the skin [23]. Thus, the antibacterial and moisturizing properties of N, O-CMCS contribute to promoting the healing of bacterial wounds. In addition, N, O-CMCS can reduce blood sugar and enhance the activity of the immune system, which has a positive effect on the treatment of diabetic bacterial wounds [23]. It is worth noting that Wei et al. found that a hydrogel treatment based on CS can control the polarization of M2 macrophages. It also has good adhesive qualities, enough mechanical strength, and the capacity to stop bleeding from wounds quickly [24]. In addition, hyaluronic acid (HA) contains a large number of hydrophilic groups, such as hydroxyl, carboxyl, etc., which provides it with a strong water absorption capacity [4]. Thus, hydrogels prepared with HA as the main component usually have ultra-water content. HA is a bioactive compound involved in the wound healing process, regulating natural healing processes such as inflammation, granulation tissue vascular remodeling, and epithelial cell migration. Therefore, HA can serve as a potential wound dressing for skin repair. In the process of wound healing, HA is an attractive candidate biomaterial that has been widely used in fields such as skin tissue engineering and regenerative medicine, skin fillers, and beauty. Chen et al. demonstrated that hydrogels containing either high- or low-molecular-weight HA considerably shortened the healing period in diabetic rats from about 70 days to 50 days. VEGF expression was increased on day 10 and CD45^+^ expression was decreased on day 3 of the HA-treated groups, respectively, suggesting it increased angiogenesis and decreased inflammation [25]. More importantly, HA is highly responsive to hyaluronidase (HAase) produced by bacteria, making it easily degraded by HAase. However, HA hydrogels alone have insufficient mechanical properties. In the present study, chemical-crosslinking grafting is commonly used to solve the problem of insufficient mechanical properties of hydrogels. Therefore, oxidized HA containing an aldehyde group (-CHO) can be obtained with the oxidation of sodium periodate (NaIO_4_) [5,26].

**Figure 1:**
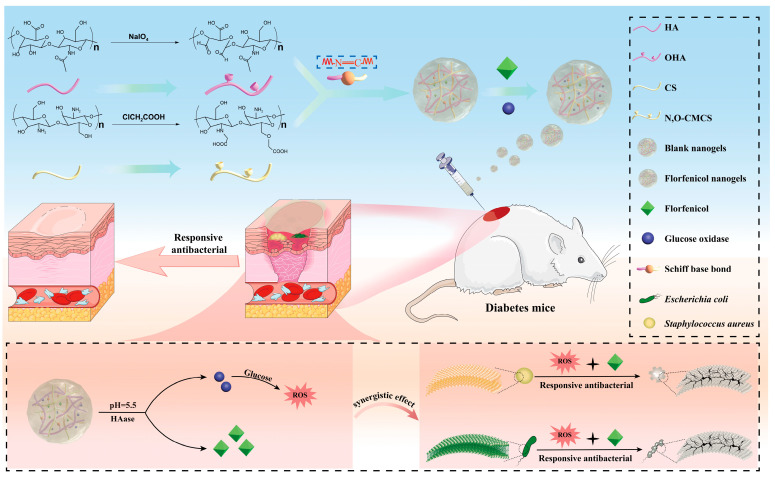
Schematic diagram of nanogel dressing with targeted glucose reduction and pH/hyaluronidase dual-responsive release for synergetic therapy of diabetic bacterial wounds.

Moreover, glucose in diabetic bacterial wounds is beneficial for bacteria to grow rapidly in a high-sugar environment. Interestingly, glucose in diabetic bacterial wounds can be catalyzed by added glucose oxidase. Once glucose is catalyzed by glucose oxidase, a large amount of reactive oxygen species (ROS) is produced, which can exert antibacterial effects. More importantly, the glucose catalyzed by glucose oxidase gradually decreases, thereby deteriorating the favorable environment for bacterial growth. The formation of Schiff’s base bond effectively limits the release of glucose oxidase and antibiotics. However, OHA may be responsive to degradation by HAase produced by bacteria in diabetic bacterial wounds. Meanwhile, the catalytic region’s pH value drops to about 5.5 due to the gluconic acid generated by the catalytic reaction and the inflammatory response brought on by bacteria (*E. coli* and *S. aureus*), which in turn causes the breakage of Schiff’s base bond, causing abundant liberation of the encapsulated active substance. Therefore, the dynamic pH responsiveness of the Schiff’s base bond, HAase responsiveness of HA, ROS, and gluconic acid produced by glucose oxidase catalysis, and targeted release of antibiotics (florfenicol) may enhance its antibacterial activity and accelerate the healing of diabetes wounds. This pH/hyaluronidase dual responsiveness helps to target the release of glucose oxidase and antibiotics (florfenicol) at diabetic bacterial wounds, thereby achieving intelligent responsive antibacterial effects (Figure 1). Given this, this study aims to obtain the antibiotic- and glucose oxidase-loaded fast-crosslinked hybrid nanogel wound dressings based on the Schiff’s base bond through N, O-CMCS (containing -NH_2_) and OHA (containing -CHO), which not only enhance the mechanical properties of the antibiotic-loaded hybrid nanogel wound dressings but also endow it with intelligent responsive antibacterial effects and synergetic therapy of diabetic bacterial wounds.

## 2. Results and Discussion

### 2.1. Synthesis of N, O-CMCS

In this study, N, O-CMCS was obtained through the carboxylation of MCAA and examined by PXRD, FTIR, ^1^H-NMR, and ^1^C-NMR. The PXRD showed that the characteristic diffraction peaks of CS (20.2°) had almost disappeared in N, O-CMCS. Correspondingly, new characteristic diffraction peaks of N, O-CMCS (27.6°, 31.9°, 45.5°, 56.4°, and 75.5°) were visible instead, which may be attributed to the successful carboxylation of CS by MCAA (Figure S1). The results of FTIR spectroscopy showed that the unique peaks for CS at 3140 cm^−1^ disappeared, as shown in the FTIR spectrum of N, O-CMCS, and were replaced by two new distinguishing peaks (3151 and 2917 cm^−1^). In addition, a symmetric stretching vibration absorption peak of COO^-^ and a characteristic carboxymethyl sodium salt absorption peak at 1428 cm^−1^ were observed in N, O-CMCS, indicating that CS underwent a carboxymethylation process. N, O-CMCS results from CS’s significant decrease in the C-O stretching vibration absorption peak of the original hydroxyl group at 1313 cm^−1^, indicating that CS added carboxymethyl groups to the N and O positions to varying degrees (Figure S2). From the ^1^H-NMR and ^1^C-NMR of CS it can be observed that δ 8.44 (s, 1H), 3.63 (s, 35H), 2.21 (s, 1H), 2.05 (s, 2H), 1.90 (s, 1H), 1.45 (s, 1H), and 1.23 (s, 2H): there were no obvious peaks in the ^1^C-NMR (Figure S3). Correspondingly, in the structure of N, O-CMCS it was found that for ^1^H NMR (400 MHz, D_2_O) δ 8.47 (s, 1H), 4.50 (s, 50H), 4.30–3.03 (m, 633H), 2.90–2.43 (m, 75H), 2.08 (s, 22H), 1.93 (s, 11H), and 1.34–1.14 (m, 19H); whilst for ^13^C NMR (101 MHz, D_2_O) δ 179.97, 178.03, 168.35, 69.51, and 61.23 was found (Figure S4). Therefore, it once again proves that N, O-CMCS was successfully synthesized through the carboxylation of MCAA. After carboxylation of MCAA, the appearance changed from clear (CS) to cloudy (N, O-CMCS); CS and N, O-CMCS exhibited the same fluidity as water in a bottle (0°, 45°, 90°, and 180°) (Figure S5). The freeze-dried samples of CS and N, O-CMCS were all in the form of white powder (Figure S6). The reconstituted solutions of CS and N, O-CMCS were all clear (solvent: 0.5% acetic acid) and transparent (solvent: ultrapure water), which proves that N, O-CMCS had excellent solubility (Figure S7). Meanwhile, SEM showed that the fresh N, O-CMCS were spherical with a smooth surface and good particle size distributions (Figure S8). The TEM of N, O-CMCS exhibited the wrinkling characteristics of polymer materials (Figure S9). The EDS of freeze-dried N, O-CMCS indicated that C, N, and O were uniformly distributed in the N, O-CMCS (Figure S10). The ZP of CS and N, O-CMCS were 30.2 ± 2.0 mV and −25.2 ± 1.2 mV, respectively (Figure S11), which may be attributed to the carboxymethyl groups. Nanoscale spherical N, O-CMCS may be more conducive for the development of drug delivery systems, thereby improving the absorption of insoluble drugs and enhancing their targeting and antibacterial activity.

### 2.2. Oxidation of HA

In this study, OHA was obtained through the oxidation of NaIO_4_ and determined by PXRD, FTIR spectroscopy, ^1^H-NMR, and ^1^C-NMR. There was no significant difference in the characteristic diffraction peaks of PXRD between HA and OHA (Figure S12), which indicated that both were in amorphous form. The presence of aldehyde groups was shown by a shoulder peak at 1730 cm^−1^ in the OHA FTIR spectrum (Figure S13). This demonstrated that NaIO_4_ effectively oxidized HA to OHA. Furthermore, the structure of OHA was determined by ^1^H-NMR and ^1^C-NMR. From the ^1^H-NMR and ^1^C-NMR of HA, it can be observed that ^1^H NMR (400 MHz, D_2_O) δ 4.49 (d, *J* = 34.4 Hz, 14H), 4.11 (d, *J* = 5.7 Hz, 4H), 3.59 (dd, *J* = 140.2, 56.6 Hz, 73H), 2.34 (d, *J* = 8.1 Hz, 4H), 2.01 (s, 21H), and 1.24 (s, 1H). There were no obvious peaks in the ^1^C-NMR (Figure S14). Correspondingly, it can be found that ^1^H NMR (400 MHz, D_2_O) δ 5.34–5.15 (m, 4H), 5.09 (d, *J* = 4.5 Hz, 5H), 4.97 (d, *J* = 12.4 Hz, 8H), 4.67 (dd, *J* = 19.2, 10.9 Hz, 7H), 4.48 (dd, *J* = 29.1, 21.5 Hz, 3H), 4.37–4.04 (m, 14H), 4.04–3.63 (m, 32H), 3.63–3.43 (m, 6H), 3.35 (d, *J* = 8.3 Hz, 2H), 2.71 (s, 1H), 2.35 (d, *J* = 7.6 Hz, 2H), 2.20–1.86 (m, 20H), and 1.23 (d, *J* = 4.1 Hz, 1H). There were no obvious peaks in the ^1^C-NMR (Figure S15). Similarly, there were no significant differences in the appearance between HA and OHA (all were clear and transparent) in a bottle (0°, 45°, 90°, and 180°) (Figure S16). The freeze-dried samples of HA and OHA were all white powder (Figure S17). The reconstituted solutions of HA and OHA were all clear and transparent, which proves that OHA had excellent solubility (Figure S18). Meanwhile, SEM showed that OHA was spherical with a smooth surface and good particle size distributions (Figure S19). The TEM of OHA exhibited that it was gelatinous (Figure S20). The EDS of freeze-dried OHA showed that C, N, and O were uniformly distributed in the OHA (Figure S21). The ZP of HA and OHA were −18.5 ± 4.9 mV and −32.8 ± 6.5 mV, respectively (Figure S22), which may be attributed to the fact that HA was successfully oxidized to OHA by NaIO_4_. Spherical OHA with nanoscale properties helps the Schiff base reaction with N, O-CMCS, thereby improving its targeting ability.

### 2.3. Formulation and Determination of Optimal Formulation

**Figure 2:**
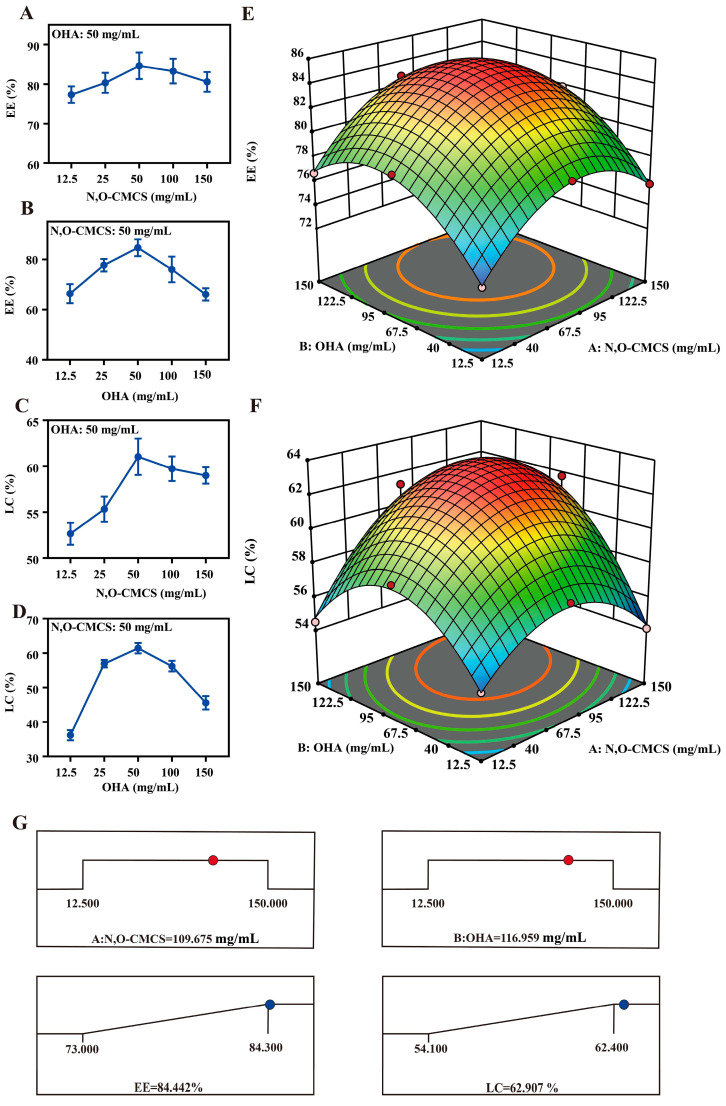
Effects of every single factor (the different concentrations of N, O-CMCS, and OHA) on EE (**A**,**B**) and LC (**C**,**D**). Three-dimensional arrangement for response surface images of the various concentrations of N, O-CMCS, and OHA to EE (**E**) and LC (**F**). (**G**) The optimal formula predicted by Design-Expert software.

The proportion of drug carriers significantly affects the LC and EE of nanoformulations. The larger the LC and EE, the greater the amount of drug encapsulated in the drug carrier and the more ideal the antibacterial activity and therapeutic effect. When LC and EE reached the maximum, the optimal formula of florfenicol nanogels was obtained. Firstly, the optimal concentration of drug carriers was screened through a single-factor experiment. The LC and EE were determined based on the standard curve of florfenicol (Figure S23). In the single-factor experiment, as the concentration of N, O-CMCS, and OHA increased, the LC and EE of florfenicol nanogels initially increased and then decreased. Figure 2A–D showed that the optimal constituent concentrations of N, O-CMCS, and OHA were 50 and 50 mg/mL, respectively. Subsequently, the concentration of N, O-CMCS (12.5, 50, and 150 mg/mL), and OHA (12.5, 50, and 150 mg/mL) were used for Box–Behnken response surface analysis [27]. By combining mathematical and statistical methods, the response surface approach calculates the relationship between the generated response surfaces and the controllable input parameters, making it easier to study and model the formulation hurdles and process parameters [28]. Thirteen experimental runs with five repeated center points were required to obtain a homogeneous estimation of the prediction variance throughout the full design space in this model. The trial design and results generated by the Design-Expert program are shown in Table S1, and the results of the thirteen groups are shown in Tables S2 and S3 along with the equation for the quadratic polynomial regression between the LC and two factors.

LC = 62.58 + 1.45 × A + 1.67 × B + 1.55× AB − 2.88 × A^2^ − 3.63 × B^2^

The quadratic polynomial regression equation between the EE and the two factors was as follows:

EE = 84.34 + 1.58 × A + 2.12 × B + 0.2500 × AB − 3.99 × A^2^ − 3.79 × B^2^

While the lack of fit was not significant (*p* > 0.05), the differences between the various treatments of the EE and LC models were extremely significant (*p* < 0.01). This suggested that the residuals were wholly the product of random errors. Both prediction models were dependent on the regression equation coefficient R^2^ and adjusted R^2^, which both show higher than 98% and reflect the range of the response values. Based on the information above, the three-dimensional reaction surface pictures were created (Figure 2E,F). Based on Design-Expert software 13.0, the optimum florfenicol nanogels were 109.675 mg/mL N, O-CMCS and 116.959 mg/mL OHA. In this instance, florfenicol was encapsulated in the greatest amount, the LC and EE were the largest, and the therapeutic effect might have been the most satisfactory. The software forecasted an LC of 62.9068% and an EE of 84.4424%, respectively. (Figure 2G). The best formulation was confirmed by creating florfenicol nanogels containing 110 mg/mL N, O-CMCS, and 117 mg/mL OHA. The synthesized florfenicol nanogels had an LC of 61.8% ± 1.5% and an EE of 83.5% ± 2.0%. Thus, the optimal florfenicol nanogels designed by the Box–Behnken response surface technique were accurate and reliable [27].

### 2.4. Characterization

**Figure 3:**
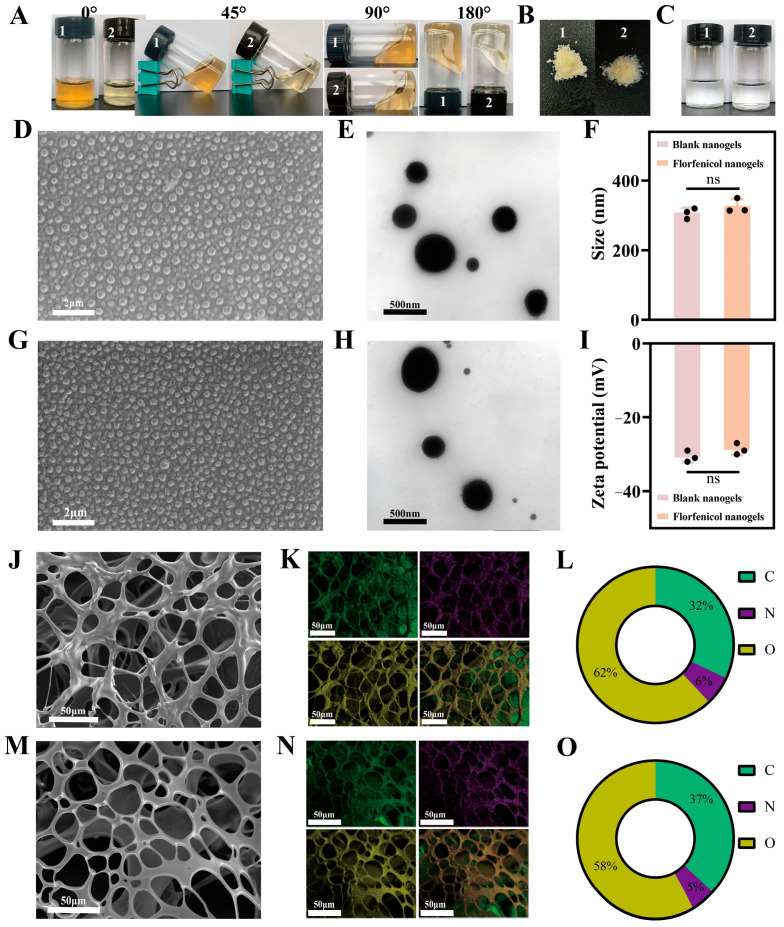
Morphological evaluation of blank nanogels and florfenicol nanogels. Appearance in a bottle (0°, 45°, 90°, and 180°) (**A**); lyophilized powder (**B**); resolvability (**C**); SEM of blank nanogels (**D**) and florfenicol nanogels (**G**); TEM of blank nanogels (**E**) and florfenicol nanogels (**H**); size distribution (**F**); zeta potential (**I**); EDS of blank nanogels (**J**,**K**) and florfenicol nanogels (**L**,**M**); and the percentage of element (**C**,**N,O**) in blank nanogels (**N**) and florfenicol nanogels (**O**). 1: blank nanogels; 2: florfenicol nanogels. (The black dots represent the three sets of data measured. ns represents no significant difference.)

The appearance (0°, 45°, 90°, and 180°), lyophilized sample, reconstituted solution, SEM, EDS, TEM, size, and ZP of blank nanogels and florfenicol nanogels are shown in Figure 3. The appearance of blank nanogels and florfenicol nanogels was dark yellow and light yellow in a bottle (0°, 45°, 90°, and 180°), respectively. Blank nanogels and florfenicol nanogels showed excellent dynamic hydrogel properties in the bottle (Figure 3A). The freeze-dried samples of blank nanogels and florfenicol nanogels were all in the form of light-yellow powder (Figure 3B). In addition, the reconstituted solutions of blank nanogels dissolved in ultrapure water and florfenicol nanogels dissolved in ultrapure water were all clear and transparent, which proved that blank nanogels and florfenicol nanogels had excellent re-solubility (Figure 3C). This indicated that florfenicol nanogels were expected to be stored by freeze-drying. Meanwhile, SEM showed that the blank nanogels (Figure 3D) and the spherical, smooth-surfaced florfenicol nanogels (Figure 3G) had good particle size distributions. The TEM of blank nanogels (Figure 3E) and florfenicol nanogels (Figure 3H) exhibited a spherical shape with a smooth surface. The mean size, ZP, and PDI of blank nanogels and florfenicol nanogels were 306.7 ± 12.5 nm, −30.7 ± 1.2 mV, and 0.13 ± 0.02 and 326.3 ± 16.7 nm, −28.7 ± 1.3 mV, and 0.18 ± 0.03, respectively (Figure 3F and Figure 3I). The nano-size is more conducive to the interaction between the prepared florfenicol nanogels and bacteria (such as *E. coli* and *S. aureus*), and thereby they play a more ideal antibacterial and antibiofilm activity. Additionally, the freeze-dried blank nanogels and florfenicol nanogels showed a three-dimensional network structure, which was highly beneficial for the transportation of drugs, oxygen, and nutrients, as well as the absorption of exudate at the wound site (Figure 3J,L). Further, the EDS of freeze-dried blank nanogels and florfenicol nanogels showed that C, N, and O were uniformly distributed in the blank nanogels and florfenicol nanogels (Figure 3K,M). Compared with the percentage of C (32%), N (6%), and O (62%) in blank nanogels (Figure 3N), the percentage of C (37%) in florfenicol nanogels increased and the percentage of N (5%) and O (58%) decreased (Figure 3O), which may be due to the addition of florfenicol. This also showed that florfenicol was successfully encapsulated into the blank nanogels, and consequently florfenicol-loaded N, O-CMCS@OHA nanogels were successfully prepared.

**Figure 4:**
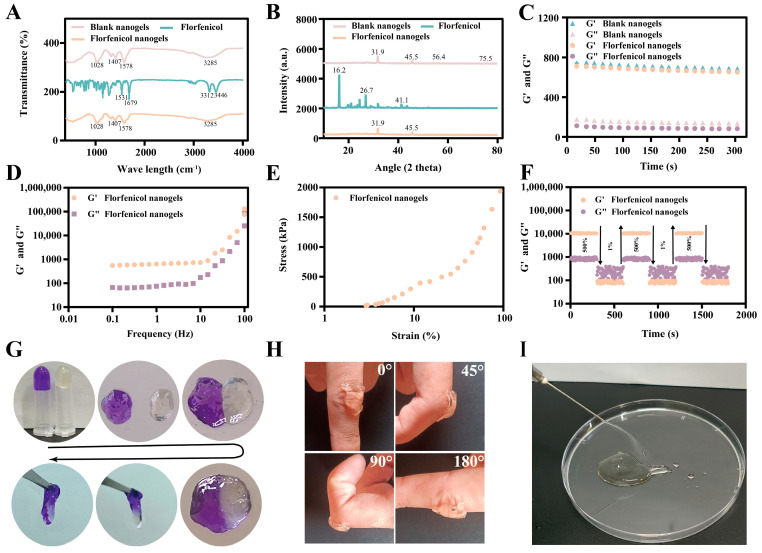
Characteristics of blank nanogels and florfenicol nanogels. FTIR (**A**); PXRD (**B**); rheological analysis of blank nanogels and florfenicol nanogels in a time sweep mode (**C**); frequency sweep tests of florfenicol nanogels for a frequency range of (0.1 to 100 Hz (**D**)); stress–strain curves of florfenicol nanogels (**E**); G′ and G″ of florfenicol nanogels in continuous step strain sweep between 1% and 500% strain at 37 °C (**F**); self-healing properties (**G**); adhesive properties (**H**); injectability (**I**).

Furthermore, the FTIR, PXRD, rheological analysis, self-healing properties, adhesiveness, injectability, and stability of blank nanogels and florfenicol nanogels are shown in Figure 4. The FTIR spectra of blank nanogels, florfenicol, and florfenicol nanogels are shown in Figure 4A. In the spectrum of blank nanogels, the characteristic peaks at 1028, 1407, 1578, and 3285 cm^−1^ were extremely similar to the characteristic peaks of florfenicol nanogels. Interestingly, when florfenicol was added to the blank nanogels to form florfenicol nanogels, its characteristic peaks (1531, 1679, 3312, and 3446 cm^−1^) had disappeared. Thus, the FTIR results indicated that florfenicol was completely encapsulated into nanogels. The PXRD characteristic diffraction peaks of blank nanogels (31.9°, 45.5°, 56.4°, and 75.5°) and florfenicol (16.2°, 26.7°, and 41.1°) are shown in Figure 4B. It is worth noting that the characteristic diffraction peaks of florfenicol had almost disappeared in florfenicol nanogels because of the formation of florfenicol-loaded N, O-CMCS@OHA nanogels (there were only two peaks: 31.9° and 45.5°). The unique diffraction peaks of the florfenicol nanogels and blank nanogels vanished, and this is likely due to Schiff’s base reaction which is created by amino groups with N, O-CMCS and aldehyde groups with OHA.

The storage modulus (G′) and loss modulus (G″) of the materials were measured throughout time at a certain frequency (1 rad/s) using a rheometer to ascertain the rheological properties of florfenicol nanogels (Figure 4C–F). It was difficult to quickly detect the junction of G′ and G″ since the florfenicol nanogels formed in a couple of seconds. G′ of the florfenicol and blank nanogels predominated during the entire duration, as seen in Figure 4C, indicating their stability. The stability of florfenicol nanogels will not be impacted by adding florfenicol. By measuring the kinetics of florfenicol nanogels, frequency scanning was used to verify the stability of the nanogels. Figure 4D illustrates that over the entire frequency range G′ predominates over G″, suggesting that florfenicol nanogels were in a dynamic hydrogel condition. The compressive stress and strain test was used to assess the elasticity of florfenicol nanogels (Figure 4E). The florfenicol nanogels were crushed at strains ranging from 1% to 100% while being squeezed externally. Once the load is lifted, they may promptly revert to their initial configuration. Furthermore, no visible damage was seen in the samples when the imposed maximum strain was 100%, suggesting that florfenicol nanogels have strong mechanical qualities and can tolerate high compression levels. G′ was smaller and lower than G″ when the strain was high (strain = 500%), as seen in Figure 4F. G′ had recovered when the strain was minimal (strain = 1%). Schiff bases of florfenicol nanogels were damaged under high strain, but they progressively recovered under low strain. The aforementioned findings demonstrated the presence of a dynamic Schiff’s base bond in florfenicol nanogels which gives them the ability to mend themselves. Furthermore, the florfenicol nanogels’ pores filled with crystal violet dye diffused into the light yellow florfenicol nanogels (which contained no crystal violet dye) in less than ten minutes, indicating that the porous-structured florfenicol nanogels had a sufficient capacity for drug loading and self-healing (Figure 4G). After florfenicol nanogels were applied to the bent fingers, florfenicol nanogels closely adhered to the fingers at the knuckle (0°, 45°, 90°, and 180°), which indicated that the prepared drug-loaded nanogel dressing (florfenicol nanogels) had excellent adhesion (Figure 4H). In addition, several use scenarios were designed to verify the feasibility of the rheological properties of florfenicol-loaded N, O-CMCS@OHA nanogels. The florfenicol nanogels were continuously injected into a culture dish through a syringe, and the water column hydrogels and “TD 413” fonts were observed, respectively (Figure 4I and Figure S24), which confirmed the good injectability of florfenicol nanogels. High temperatures, high humidity, and high light did not substantially alter the size, ZP, or PDI of florfenicol nanogels according to the effect factors test (Table S4). It implied that one may perhaps predict the storage stability of florfenicol nanogels. Finally, it was shown how florfenicol nanogels might be used in practice as a wound dressing. The PBS, glucose oxidase, and florfenicol nanogels were added to the glucose solution. After 12 h, the glucose concentrations decreased to 13.8 ± 0.3, 7.5 ± 0.3, and 7.3 ± 0.1 mmol/L, respectively (Figure S25). This result confirmed that florfenicol nanogels can effectively reduce glucose concentration.

### 2.5. pH/HAase Dual-Responsive Performances

**Figure 5:**
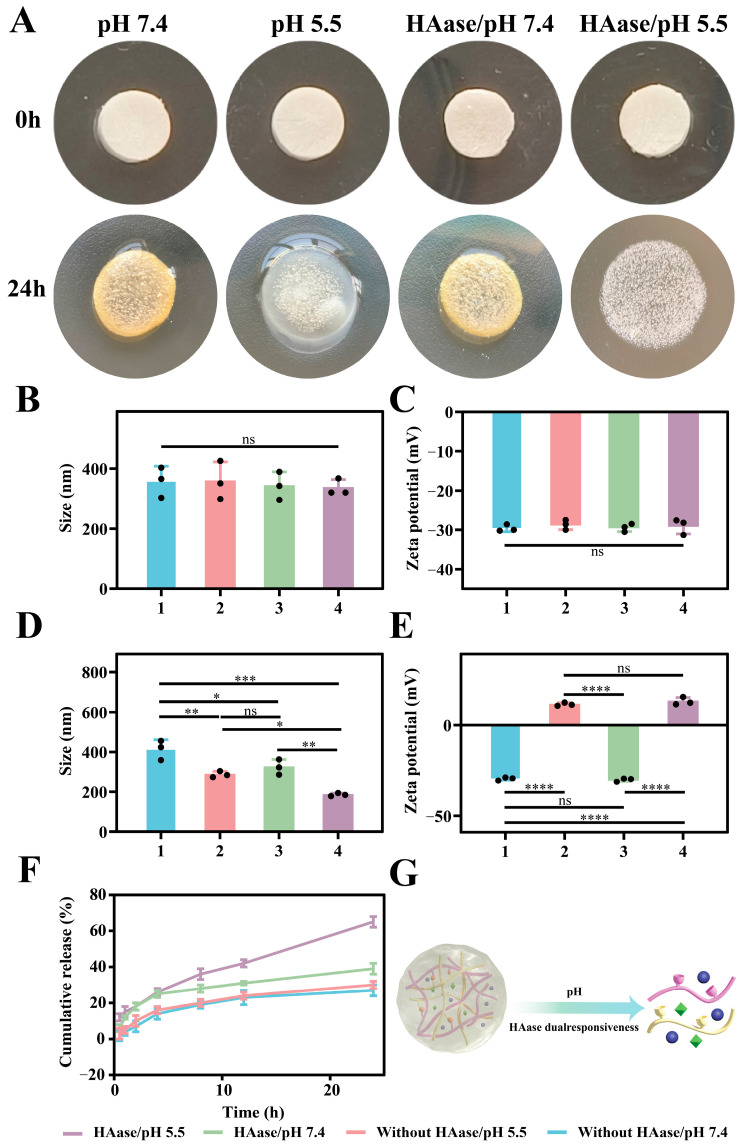
Improving pH/hyaluronidase dual responsiveness release of florfenicol nanogels. (**A**) Morphology change of florfenicol nanogels in different microenvironments (pH 7.4 without HAase; pH 5.5 without HAase; pH 7.4 with HAase; pH 5.5 with HAase); size and ZP of florfenicol nanogels in different microenvironments at 0 h (**B**,**C**) and 24 h (**D**,**E**); in vitro release of florfenicol nanogels in different microenvironments (**F**); and the hydrolysis mechanism of florfenicol nanogels (**G**). (* *p* < 0.05, ** *p* < 0.01, *** *p* < 0.001 and **** *p* < 0.0001; The black dots represent the six sets of data measured. ns represents no significant difference.)

Considering that Schiff’s base bond and N, O-CMCS have pH-responsiveness, and OHA has HAase-responsiveness, florfenicol nanogels may have pH/HAase dual responsiveness to quickly release florfenicol when dressing the wound. Thus, florfenicol nanogels were put in different microenvironments (pH: 7.4 without HAase; pH: 5.5 without HAase; pH: 7.4 with HAase; and pH: 5.5 with HAase), and the morphology was examined at 0 and 24 h. At 24 h, florfenicol nanogels changed from white to yellow at pH 7.4. However, there was no significant color change at pH: 5.5, which may be due to differences in the pH. In addition, under the action of HAase and pH: 5.5, florfenicol nanogels had obvious swelling (Figure 5A). At 0 h, the particle size and ZP of the florfenicol nanogels were ≈330 nm (Figure 5B) and ≈−29 mV, respectively (Figure 5C). At 24 h, the particle sizes of the florfenicol nanogels were 412.8 ± 40.0 nm (pH: 7.4 without HAase), 287.6 ± 12.7 nm (pH: 5.5 without HAase), 324.3 ± 31.0 nm (pH: 7.4 with HAase), and 185.3 ± 6.6 nm (pH: 5.5 with HAase) (Figure 5D). This suggested that under acidic conditions, the nanogels may undergo shrinkage or disintegration. Upon the introduction of HAase into the nanogels its particle size was further reduced, confirming that the nanogels’ structure could be effectively degraded by Haase and thereby enhancing its potential for environmentally responsive drug release. The ZP of the florfenicol nanogels was −29.5 ± 0.7 mV (pH 7.4 without HAase), 11.5 ± 0.7 mV (pH 5.5 without HAase), −30.1 ± 0.8 mV (pH 7.4 with HAase), and 13.1 ± 1.7 mV (pH 5.5 with HAase) (Figure 5E). It was also demonstrated that under acidic conditions the surface charge of the nanogels was reversed, which may be attributed to protonation and HAase-mediated degradation. Furthermore, the additional increase in ZP induced by HAase at pH 5.5 suggested that positively charged groups were exposed upon enzymatic cleavage. The changes in particle size and potential are similar to previous reports [29]. The environmental pH/HAase dual responsiveness was calculated by estimating the release of the florfenicol nanogels in PBS (pH 5.5/7.4) with and without HAase at 37 ± 0.5 °C, taking into account the microenvironment (pH 5.5 and HAase) of diabetic bacterial wounds. After 24 h, approximately 27.2% ± 3.1% of the florfenicol nanogels were released at pH 7.4 without HAase, as opposed to 39.1% ± 3.0% that were released at pH 7.4 with HAase. Additionally, 30.1% ± 2.1% of the florfenicol nanogels were released at pH 5.5 without HAase, compared with 64.9% ± 3.1% that were released at pH 5.5 with HAase (Figure 5F). These findings indicated that the composite nanogels displayed pH/hyaluronidase dual responsiveness and on-demand release performance after dressing on the wound because of the pH-responsiveness of Schiff’s base reaction and N, O-CMCS, and the HAase-responsiveness of OHA. This might be because strongly protonated amino groups at pH 5.5 repel one another, creating holes that let water molecules enter and grow into the nanogels’ cores. At the same time, due to OHA in the role of HAase, the florfenicol nanogels had been split. On the contrary, at higher pH values (pH: 7.4) florfenicol nanogels with low protonation result in smaller swelling. Thus, the split of florfenicol nanogels was higher under the action of HAase and pH: 5.5. This may be attributed to the swelling of florfenicol nanogels acted on by HAase and pH: 5.5, which allows a large number of water molecules to enter the florfenicol nanogels for swelling. However, in the other three microenvironments (pH: 7.4 without HAase; pH: 5.5 without HAase; pH: 7.4 with HAase), the swelling of florfenicol nanogels is small. Therefore, florfenicol nanogels exhibited an obvious dual pH- and HAase-responsiveness and can achieve the on-demand release (Figure 5G).

### 2.6. Antibacterial Activity

**Figure 6:**
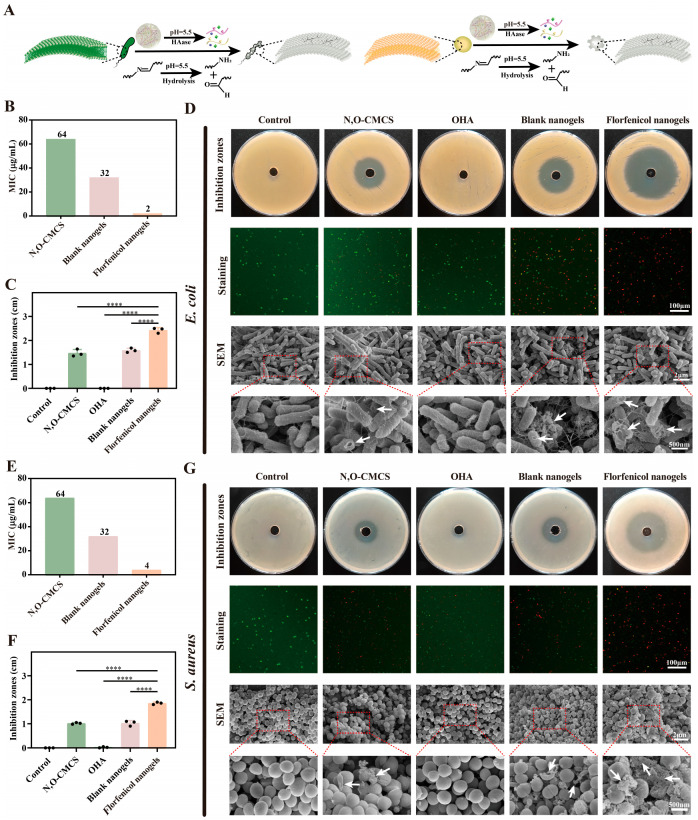
The antibacterial effect of N, O-CMCS, OHA, blank nanogels, and florfenicol nanogels for *E. coli* and *S. aureus* treatments. (**A**) Schematic diagram of the antibacterial mechanism based on florfenicol nanogels. The MICs, inhibition zones, live/dead bacterial staining, SEM image of N, O-CMCS, OHA, blank nanogels, and florfenicol nanogels against *E. coli* (**B**–**D**) and against *S. aureus* (**E**–**G**). (**** *p* < 0.0001; The black dots represent the three sets of data measured.)

The antibacterial activity of N, O-CMCS, OHA, blank nanogels, and florfenicol nanogels against *E. coli* and *S. aureus* is shown in Figure 6. *E. coli* had MICs of 64, >128, 32, and 2 µg/mL for N, O-CMCS, OHA, blank nanogels, and florfenicol nanogels, while *S. aureus* had MICs of 64, >128, 32, and 4 µg/mL (Figure 6B,E). Compared to N, O-CMCS, OHA, and blank nanogels, florfenicol nanogels showed higher antibacterial activity against *E. coli* and *S. aureus*. This might be connected to the florfenicol nanogels’ long-lasting demand-release action created by Schiff’s base bond and the synergistic effect of florfenicol and N, O-CMCS. In a three-dimensional net construction, florfenicol may be released constantly and yet show superior antibacterial activity in terms of quantitative data. These might be explained by the florfenicol nanogels’ extended half-life (the time required for the concentration of nanogels to decrease by half) and higher drug concentration. Meanwhile, the inhibition zones of N, O-CMCS, OHA, blank nanogels, and florfenicol nanogels were 1.47 ± 0.12, 0, 1.58 ± 0.07, and 2.42 ± 0.09 cm for *E. coli* (Figure 6C) and 1.02 ± 0.03, 0.02 ± 0.02, 1.02 ± 0.09, and 1.85 ± 0.04 cm for *S. aureus*, respectively (Figure 6F). Compared with N, O-CMCS, OHA, and blank nanogels, florfenicol nanogels had larger inhibition zones. Furthermore, the mixture of *E. coli* or *S. aureus* and N, O-CMCS, OHA, blank nanogels, and florfenicol nanogels (the doses were all matching 1×MIC) was treated using the live/dead bacterial staining kit (Figure 6D,G). Florfenicol nanogels also exhibited higher bactericidal activity than N, O-CMCS, OHA, and blank nanogels, as evidenced by the findings’ increased number of living (colored red) and decreased number of dead (colored green) bacterial cells. The findings from the inhibition zones and MICs were consistent with these results. Interestingly, compared to *S. aureus*, *E. coli* was more susceptible to the antibacterial effects of florfenicol nanogels. This might be a result of *S. aureus*’s thicker cell wall. Compared with *E. coli*, florfenicol nanogel finds it difficult to destroy the cell membrane and cell wall of *S. aureus*. To verify this hypothesis, SEM was used to observe *E. coli* or *S. aureus* treated with N, O-CMCS, OHA, blank nanogels, and florfenicol nanogels, as well as physiological saline (the control group). Compared with the control group, OHA had no significant effect against *E. coli* and *S. aureus*. However, the action of N, O-CMCS, blank nanogels, and florfenicol nanogels had significant effects against *E. coli*, damaging the bacterial cell wall and membrane, which is why the holes emerged. The pores in the group of florfenicol nanogels were particularly noticeable. At the same time, the bacteria also experienced a significant rupture and their contents spilled out, which might mean that the florfenicol nanogels have harmed the bacteria’s cell wall and membrane. Similarly, *S. aureus* treated with florfenicol nanogels also broke, became slightly larger, and the contents inside the bacteria flowed out. This may be due to the destruction of the bacterial cell wall, allowing a large amount of water to enter the bacteria through the bacterial cell membrane. Subsequently, the bacteria began to swell and rupture, causing the contents to be released (Figure 6A). In comparison to N, O-CMCS, OHA, and blank nanogels, florfenicol nanogels have greater antibacterial activity against both Gram-positive and Gram-negative bacteria, which is related to the synergistic and new antibacterial mechanism produced by florfenicol nanogels. This new and synergistic mechanism mainly includes the fact that florfenicol nanogels promote the rapid production of ROS and that the excessive ROS will destroy the bacterial biofilm, thereby increasing the permeability of florfenicol nanogels to the biofilm and their activity against bacteria. It is worth noting that the oxidative damage caused by ROS to bacteria does not target certain bacterial metabolic stages, which will lessen the likelihood of bacterial resistance developing. More and more evidence confirms that cell membrane damage is linked to ROS-induced oxidative stress damage, which is an efficient antibacterial mechanism. After florfenicol nanogel treatment, the endogenous ROS level of bacteria increased significantly (Figure S26). Therefore, florfenicol nanogels can serve as an effective antibacterial agent by inducing ROS bursts to inhibit the growth of bacteria.

### 2.7. Evaluation of In Vivo Wound Healing

**Figure 7:**
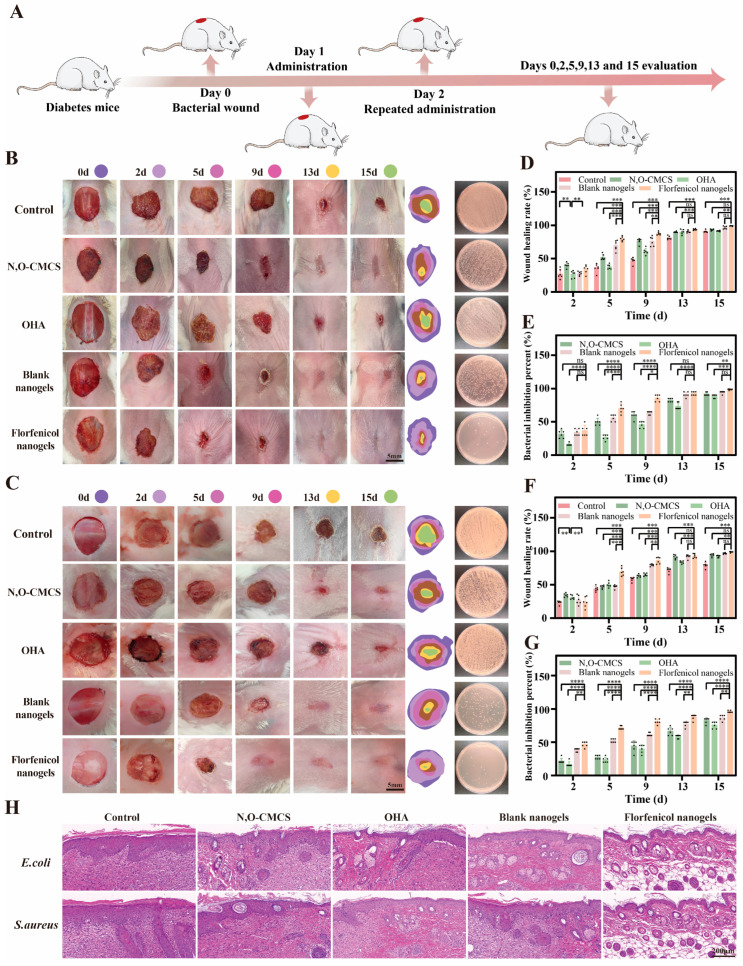
In vivo diabetic bacterial wound healing studies. (**A**) Treatment schedules. Representative images of *E. coli* (**B**) or *S. aureus* (**C**) diabetic bacterial wounds in different treatment groups (control, N, O-CMCS, OHA, blank nanogels, and florfenicol nanogels) on days 0, 2, 5, 9, 13, and 15. Corresponding wound healing rate of *E. coli* (**D**) or *S. aureus* (**F**) on days 0, 2, 5, 9, 13, and 15. Corresponding bacterial inhibition percentage of *E. coli* (**E**) or *S. aureus* (**G**) on days 0, 2, 5, 9, 13, and 15. (**H**): Histological evaluation. (* *p* < 0.05, ** *p* < 0.01, *** *p* < 0.001 and **** *p* < 0.0001; The black dots represent the six sets of data measured. ns represents no significant difference.)

The healing rate of each group in the *E. coli*- and *S. aureus*-infected diabetic wound models was monitored to assess the impact of florfenicol nanogels on infected wound healing. In Figure 7A, the treatment regimens are displayed. The treatments were divided into the following five groups: blank nanogels, florfenicol nanogels, OHA, N, O-CMCS, and the control (physiological saline). Figure 7 displays typical photos of diabetic bacterial wounds infected with *E. coli* (Figure 7B) and *S. aureus* (Figure 7C) at various treatment stages of the wound. Remarkably, on day 2, significant abscesses were seen in the control group’s wounds infected with *S. aureus* and *E. coli*. These sores remained untreated until the thirteenth day. In contrast, the florfenicol nanogels group had a small abscess, which may have been caused by their strong antibacterial activity. Furthermore, on day nine, there were already extremely few abscesses, which were difficult to see, in the florfenicol nanogels groups. Significantly better wound healing manifestations were observed on days 2 and 5 when the florfenicol nanogels group was compared to the N, O-CMCS, OHA, and blank nanogels groups. Up to the end of the fifteenth day, the wound healing of the florfenicol nanogels group had advanced more quickly. In quantitative analysis, the wounds in the florfenicol nanogels group almost acquired complete healing (*E. coli*: 99.1 ± 0.2%; *S. aureus*: 99.0 ± 0.7%), whilst the wounds were still unhealed in the control group (*E. coli*: 90.8 ± 1.6%; *S. aureus*: 79.2 ± 3.7%), N, O-CMCS group (*E. coli*: 93.3 ± 1.1%; *S. aureus*: 94.1 ± 2.0%), OHA group (*E. coli*: 91.4 ± 0.6%; *S. aureus*: 92.4 ± 1.5%), and blank nanogels group (*E. coli*: 96.5 ± 1.9%; *S. aureus*: 96.8 ± 0.9%) on day 15 (Figure 7D,E). These results showed that the group that received florfenicol nanogels had the quickest rate of diabetic wound healing out of all the groups. The percentage of bacterial inhibition at the diabetic bacterial wound site is a reliable measure of the wound dressing material’s antibacterial efficacy. Bacterial load samples in the wound region were matched across different groups on days 2, 5, 9, 13, and 15. On day 15, the florfenicol nanogels group outperformed the N, O-CMCS group (*E. coli*: 91.7 ± 2.3%; *S. aureus*: 82.5 ± 3.8%), OHA group (*E. coli*: 88.3 ± 2.3%; *S. aureus*: 75.8 ± 4.5%), and blank nanogels group (*E. coli*: 94.0 ± 1.9%; *S. aureus*: 86.7 ± 3.7%). This was because the florfenicol nanogels group showed the highest bacterial inhibition percent (*E. coli*: 98.5 ± 0.8%; *S. aureus*: 96.0 ± 1.4%), which indicated a better antibacterial effect (Figure 7D,E). The difference in bacterial inhibition percentage amongst the control, N, O-CMCS, OHA, blank nanogels, and florfenicol nanogels groups can be related to the quick release of florfenicol in the florfenicol nanogels matrix. Thus, the quantitative analysis further confirmed that the florfenicol nanogels displayed better antibacterial effects in comparison to the control, N, O-CMCS, OHA, and blank nanogels on day 15.

Numerous intricate biological mechanisms, including bacterial infection, are involved in the diabetic bacterial wound healing process. The progress of diabetic bacterial wound healing was assessed by histological evaluation of diabetic bacterial wound tissues in different treatment groups (control, N, O-CMCS, OHA, blank nanogels, and florfenicol nanogels) in order to confirm the effect of the drug-loaded nanogel dressing on the aforementioned processes. Numerous inflammatory cells were found in the dermal tissues of the control group according to H&E staining, and the inflammatory cells were decreased following the application of blank nanogels. Compared to the other treatments (control, N, O-CMCS, OHA, and blank nanogels group), the inflammatory cells were nearly invisible in the florfenicol nanogels treatment group. Additionally, new structures that resembled hair follicles were observed, along with some epithelial cells and connective tissues that were more neatly arranged. Thus, the prepared dressings showed satisfactory therapeutic effects for the full-thickness mouse skin defect wounds.

## 3. Conclusions

In this study, florfenicol-loaded N, O-CMCS@OHA nanogels were successfully prepared by Schiff’s base reaction between OHA-containing aldehyde groups and N, O-CMCS-containing amino groups. The florfenicol nanogels exhibited obvious pH- and HAase-responsiveness to achieve efficient release in the microenvironment (pH 5.5 with HAase) of diabetic bacterial (*E. coli* and *S. aureus*) wounds. Moreover, florfenicol nanogels accelerated the healing of diabetic bacterial wounds and demonstrated synergetic antibacterial efficacy. The developed dressing performed better in hastening the healing of diabetic bacterial wounds because they are a superior wound dressing with synergetic targeted glucose reduction and pH/hyaluronidase dual-responsive release.

## 4. Materials and Methods

### 4.1. Materials

CS with a molecular weight (MW) of 161.16 KDa, viscosity of 50–800 mPa.s, and degree of acetylation of 80–95%, monochloroacetic acid (MCAA; MW: 94.50 Da), HA (MW: 403.3 kDa, 97.0%), NaIO_4_ (99.9%), isopropyl alcohol (≥99.5%; MW: 60.01 Da), hyaluronidase, and ethylene glycol (content: 99.9%) were purchased from Dingyuan Biotechnology Co., Ltd. (Alar, China). Florfenicol-active pharmaceutical ingredients (content: 98%) were obtained from Macklin (Shanghai, China). Tryptone Soya Broth (TSB), 2′,7′-dichlorofluorescein-diacetate (DCFH-DA), and phosphate-buffered saline (PBS) were provided by Pumoke Biotechnology Inc. (Wuhan, China), along with the antibodies used for the live/dead bacterial staining investigation. The *E. coli* and *S. aureus* were offered by the Engineering Laboratory for Tarim Animal Diseases Diagnosis and Control (Alar, China). A Milli-Q system was used to prepare the water (Millipore, Burlington, MA, USA). The remaining reagents in the text were either equal or of analytical grade.

### 4.2. Synthesis of N, O-CMCS

The synthesis of N, O-CMCS was conducted according to previously published methods [23]. In short, 75 milliliters of isopropyl alcohol were mixed with 10 g of CS and swirled at 25 °C. After that, the mixture was mixed with 25 mL of a 10 mol/L sodium hydroxide aqueous solution, which was added every five minutes in five equal parts. At 800 RPM, the alkaline suspension was constantly stirred for 30 min. Subsequently, 4 g MCAA was added in drops every 5 min for a total of 5 times (20 g MCAA). The whole reaction system was controlled at 60 °C and stirred for 3 h. The reaction mixture was filtered to provide the solid product of N, O-CMCS, which was then extensively washed three times with 80% *v*/*v* ethanol solution and multiple times with 100% ethanol. A lyophilizer (FDU-1200, Tokyo, Japan) produced the dry product of N, O-CMCS. The lyophilized N, O-CMCS was determined by powder X-ray diffraction (PXRD) (D8 Advance, Bruker, Munich, Germany), proton nuclear magnetic resonance spectroscopy (^1^H-NMR and ^1^C-NMR) (Varian 400 spectrometer, Varian Inc, Palo Alto, CA, USA), and Fourier transform infrared (FTIR) spectroscopy (Nicolet iS50, Thermo Scientific Inc., Waltham, MA, USA). Furthermore, the substitution degree of N, O-CMCS was measured by a potentiometric titrator (ZDJ-4A, Shanghai Yidian Scientific Instrument Co., Ltd., Shanghai, China). The CS dissolved in 0.5% acetic acid and N, O-CMCS dissolved in ultrapure water were placed in a bottle (0°, 45°, 90°, and 180°) to observe their fluidity, respectively. Simultaneously, the morphology of fresh N, O-CMCS was observed by scanning electron microscopy (SEM) APREO, Thermo Scientific Inc., USA, and transmission electron microscopy (TEM), JEM-2100Plus, Japan. The morphology of the freeze-dried N, O-CMCS was observed by SEM, and elemental analysis was determined by energy dispersive spectroscopy (EDS), X-Max N 150; Oxford; UK. The zeta potential (ZP) of N, O-CMCS was measured by a Zetasizer ZX3600 (Malvern Instruments, Malvern, UK). Finally, CS and N, O-CMCS were dissolved in 0.5% acetic acid and ultrapure water again to compare their resolvability, respectively.

### 4.3. Oxidation of HA

By oxidizing HA with NaIO_4_, OHA was produced with previously described procedures [15]. In short, 100 mL of ultrapure water was mixed with 0.5 g of HA, which was dissolved at 70 °C. To prepare a NaIO_4_ solution, 0.25 g of NaIO_4_ was simultaneously added to 10 mL of ultrapure ddH_2_O. The HA solution was then mixed with the NaIO_4_ solution dropwise for 24 h at 800 RPM while the mixture was left in the dark. To halt the oxidation reaction, 3 mL of ethylene glycol was added and agitated for 1 h at 800 RPM. For three days, the mixture was subjected to excessive dialysis in a dialysis bag (MW: 3500) to eliminate unreactive species. The mixture was then lyophilized to produce a whitish powder. The conversion of OHA was confirmed by FTIR spectroscopy, PXRD, and NMR (^1^H-NMR and ^1^C-NMR). The HA dissolved in ultrapure water and OHA dissolved in ultrapure water were placed in a bottle (0°, 45°, 90°, and 180°) to observe their fluidity, respectively. Simultaneously, the fresh morphology of OHA was detected by SEM and TEM. On the other hand, the morphology of the freeze-dried OHA was observed by SEM, and EDS determined the elemental analysis. The ZP of OHA was measured by using a Zetasizer ZX3600 at 25 °C. Finally, the HA and OHA were dissolved in ultrapure water again to compare their resolvability.

### 4.4. Formulation of Florfenicol-Loaded N, O-CMCS@OHA Nanogels

The florfenicol-loaded N, O-CMCS@OHA nanogels were formulated by Schiff’s base reaction [17]. Briefly, lyophilized OHA (12.5, 25, 50, 100, or 150 mg) was added to 1 mL of ultrapure water with stirring to dissolve. Simultaneously, lyophilized N, O-CMCS (12.5, 25, 50, 100, or 150 mg) was also added to 1 mL of ultrapure water and stirred. Subsequently, the OHA solution was added dropwise to the N, O-CMCS with magnetic stirring at 800 RPM, obtaining N, O-CMCS@OHA nanogels (blank nanogels). Then, 0.2 g florfenicol was dissolved in 200 μL dimethylformamide to obtain the florfenicol solution. Finally, the florfenicol solution and 1 mg glucose oxidase were added to blank nanogels under magnetic stirring at 800 RPM to form the florfenicol-loaded N, O-CMCS@OHA nanogels (florfenicol nanogels). The standard curve of florfenicol concentrations, ranging from 1 to 1000 µg/mL, was plotted using an ultraviolet spectrophotometer. The optimal concentrations of N, O-CMCS, and OHA were evaluated by loading capacity (LC) and encapsulation efficiency (EE). Each sample was formulated in triplicate. The data are expressed as the mean ± SD.

### 4.5. Single-Factor Experiment

With the concentrations of OHA and N, O-CMCS as the variables, and LC and EE as the evaluation index, the optimal concentrations were screened. In brief, the concentrations of OHA (50 mg/mL) were fixed to investigate the impact of the amount of N, O-CMCS (12.5, 25, 50, 100, or 150 mg/mL) on the LC and EE. Similarly, the concentrations of N, O-CMCS (50 mg/mL) were fixed to explore the effect of the OHA concentrations (12.5, 25, 50, 100, or 150 mg/mL) on the LC and EE.

### 4.6. Box–Behnken Response Surface Analysis

Again, the Design-Expert 8.0 software (State-Ease, Inc., Minneapolis, MN, USA) accurately determined the optimal N, O-CMCS, and OHA concentrations. At the same time, the EE and LC were used as assessment indices. The elements and levels of the Box–Behnken design were shown in Table S1.

### 4.7. Physicochemical Characterization

#### 4.7.1. Surface Morphology

The appearance, lyophilized sample, reconstituted solution, SEM, and TEM were utilized to characterize the surface morphology of blank nanogels and florfenicol nanogels. Briefly, the freshly prepared blank nanogels and florfenicol nanogels were placed in a bottle (0°, 45°, 90°, and 180°) to observe their gel state, respectively. Subsequently, these freshly prepared samples were placed in a lyophilizer for freeze-drying to obtain lyophilized samples, and the surface morphology of the lyophilized powder samples was observed. In addition, the lyophilized samples were re-dissolved in ultrapure water to obtain a reconstituted solution for evaluating their solubility. Furthermore, blank nanogels and florfenicol nanogels were diluted one-hundred times and put into the ultrasonic cleaner (BILON3-120A, Shanghai, China) to be ultrasonicated for 30 min. Two microliters of the solution were then applied on a silicon wafer. The silicon wafer samples were dried in an oven, then coated with gold using ion sputtering and sent through a SEM at a voltage of 20 kV. Additionally, the cross sections of the freeze-dried N, O-CMCS, OHA, blank, and florfenicol nanogels were exposed by cutting them, respectively. SEM was used to examine the morphology of the freeze-dried samples, and EDS was used to assess the elemental analysis. A TEM was used to analyze the morphology of the blank and florfenicol nanogels after they were both deposited in thin-sliced copper grids and allowed to dry at room temperature using sodium phosphotungstate (2%) negative staining [17].

#### 4.7.2. The Mean Size, ZP, and Polydispersity Index (PDI)

The mean size, ZP, and PDI of blank nanogels and florfenicol nanogels were measured using a Zetasizer ZX3600 at 25 °C [17].

#### 4.7.3. PXRD and FTIR

The PXRD and FTIR of CS, N, O-CMCS, HA, OHA, blank nanogels, and florfenicol nanogels were analyzed using an X-ray diffractometer and an FTIR spectrophotometer after they were freeze-dried, respectively [17].

#### 4.7.4. Rheological Analysis

Using a parallel plate (P20 TiL), rheological measurement of the blank and florfenicol nanogels was performed using a HAAKE MARS RS6000 rheometer (Thermo Scientific, Bremen, Germany). The samples were placed on a level plate, and the borders of the parallel plate were sealed with silicone oil. A temporal sweep test was used to measure the hydrogels’ loss modulus (G″) and storage modulus (G′). To assess the viscoelasticity and investigate the connection between the modulus value and the external stress, samples were produced in a similar manner. The test was conducted at a predetermined speed of 100 mm/min in order to determine the compression characteristics, with the compressive strain going up to 100%. Last but not least, the florfenicol nanogels were regularly exposed to an oscillating strain of 1% and 500% every 800 s in order to evaluate their capacity to recover from strain deformation [22].

#### 4.7.5. Self-Healing Properties

Two portions of florfenicol nanogels were prepared, one of which was dyed with purple dye and then placed in a centrifuge tube. The other florfenicol nanogels were used as the control without treatment. Two portions of samples were contacted at 37 °C for 10 min to observe the fusion of florfenicol nanogels dyed with purple dye and untreated florfenicol nanogels to evaluate their dynamic healing [17].

#### 4.7.6. Adhesiveness

To assess the adhesiveness of the florfenicol nanogels after they were applied to the bent fingers, the status of the joint at various angles (0°, 45°, 90°, and 180°) was used [5].

#### 4.7.7. Injectability

The florfenicol nanogels were placed in the syringe and the injectability was evaluated by observing the state of the injected florfenicol nanogels [5].

#### 4.7.8. Stability

Influencing factor studies, involving increased temperature, intense light, and high humidity, were used to assess the stability of florfenicol nanogels. After being placed in a container, the florfenicol nanogels were exposed to 40 °C for a high-temperature test, 90% ± 5% for a humidity test, and 4500 ± 500 l× for illumination for ten days, respectively. On the fifth and tenth days, samples were photographed and subsequently the changes in size, ZP, and PDI of samples were measured using a Zetasizer ZX3600 [30].

#### 4.7.9. Reduce Glucose Performance

The PBS, glucose oxidase, and florfenicol nanogels were added to a glucose solution (15 mmol/L), and the glucose concentrations of each group were measured after 12 h.

### 4.8. pH/hyaluronidase Dual-Responsive Release

In this study, the responsive release of florfenicol nanogels in different microenvironments (pH: 7.4, absence of HAase; pH: 5.5 without HAase; pH: 7.4 with HAase; and pH: 5.5 with HAase) was studied. Briefly, approximately 0.1 g of freeze-dried CS, N, O-CMCS, HA, OHA, blank nanogels, and florfenicol-loaded N, O-CMCS@OHA nanogels were immersed in 10 mL PBS (pH 5.5/7.4) with or without 80 μg/mL HAase at 37 °C. At 0, 0.5, 1, 2, 4, 8, 12, and 24 h, samples were photographed and subsequently the changes in size and ZP of samples were measured using a Zetasizer ZX3600. Then, the concentrations of florfenicol released in vitro were measured using a UV-Vis spectrophotometer (PerkinElmer, Lambda 365, Shelton, CT, USA). Finally, the in vitro release curves of florfenicol nanogels in different microenvironments were plotted following the cumulative release percentage [17].

### 4.9. In Vitro Antibacterial Activity Studies

#### 4.9.1. Broth Macrodilution Method

The minimum inhibitory concentrations (MICs) of N, O-CMCS, OHA, blank nanogels, and florfenicol nanogels against *E. coli* and *S. aureus* were studied using the broth microdilution method, as recommended by the Clinical and Laboratory Standards Institute (CLSI) [16]. Briefly, N, O-CMCS, OHA, blank nanogels, and florfenicol nanogels were serially diluted in TSB. To reach a final bacterial concentration of 1 × 10^6^ CFU/mL, *E. coli* and *S. aureus* were then employed for each tube. As a control, physiological saline was employed. Following a 24 h incubation period at 37 °C, the lowest concentration that inhibited the observable growth of bacteria was identified.

#### 4.9.2. Inhibition Zones

The estimated inhibitory zones of N, O-CMCS, OHA, blank nanogels, and florfenicol nanogels against *S. aureus* and *E. coli* were discovered. An aseptic plate was filled with 15 mL of agar medium, and after the agar solidified 5 mL of agar media was added along with 0.1 mL of bacterial fluid containing 1 × 10^6^ CFU/mL of either *E. coli* or *S. aureus.* After it had been set, holes were made in the firm agar using a proper straw. Next, 50 µL N, O-CMCS, OHA, blank nanogels, and florfenicol nanogels were added. As a control, physiological saline was employed. The size of the inhibitory zones was measured and recorded after the *S. aureus* or *E. coli* cultures were cultured for 24 h. at 37 °C in an incubator with 5% CO_2_.

#### 4.9.3. Live/Dead Bacterial Staining Analysis

Separately, the N, O-CMCS, OHA, blank, and florfenicol nanogels were combined with *S. aureus* and *E. coli*. All of the samples were processed with the live/dead backlight bacterial viability kit following a two-hour incubation period. Eventually, a laser confocal microscope (A1RHD25&-SIM, Nikon, Tokyo, Japan) was used to analyze the bacterial suspensions using 5 μL which had been put onto a slide. Physiological saline was used as a control in this study.

#### 4.9.4. Morphological Analysis

This work employed SEM to investigate *E. coli* and *S. aureus* treated with blank nanogels, florfenicol nanogels, N, O-CMCS, OHA, and physiological saline (the control group). In summary, N, O-CMCS, OHA, blank nanogels, and florfenicol nanogels (all 1 × MIC) were used to culture *E. coli* or *S. aureus* (1 × 10^6^ CFU/mL) in triplicate on a cover glass for 24 h at 37 °C. Following that, the bacteria were healed and dried out. Briefly, for 2 h the samples were fixed at 4 °C using 2.5% glutaraldehyde. Following two 15 min surface washing sessions, the samples were dried for a total of 20 min using ethanol concentrations of 30%, 50%, 70%, 90%, 95%, and 100%. Finally, each sample was examined by SEM after critical point drying and gold sputter coating.

#### 4.9.5. Bacterial ROS Level Test

To find out whether bacteria were producing ROS, a DCFH-DA probe was employed. In summary, blank nanogels, florfenicol nanogels, OHA, and N, O-CMCS were combined with 1 mL of bacterial solution (1 × 10^6^ CFU/mL) and incubated for 2 h at 37 °C. The control in this study was physiological saline. After that, the treated bacteria (*E. coli* or *S. aureus*) were cultivated with 10 µM DCFH-DA (Ex: 488 nm/Em: 525 nm) for 40 min while being shielded from the light. The bacteria were stained and then collected by centrifugation (3000 r/min, 5 min) and three rounds of washing to ensure there were no remaining extra fluorescent probes. The fluorescence imaging and fluorescence intensity detection of bacteria were performed using an enzyme-linked immunosorbent analyzer and a laser confocal microscope, respectively.

### 4.10. In Vivo Wound Healing

Wuhan Pinuofei Biological Co., Ltd. (Wuhan, China) provided sixty male Kunming mice, 6–8 weeks old and weighing 18–20 g. Following the guidelines established by the Animal Science Academy of Tarim University’s Ethics Committee, the Institutional Animal Care and Use Committee at Tarim University approved the study’s animal approach and all experimental protocols involving the handling of mice (approved number: 2023008). Prior to surgery, the dorsal hair of every mouse was shaved. A full-thickness incision was made on each mouse’s back after they were sedated. Following this, 40 μL of each of the two bacterial suspensions (1 × 10^8^ CFU/mL) was introduced to the location of the wound. Five groups of six mice each were created by randomization, forming the control group, N, O-CMCS group, OHA group, blank nanogels group, and florfenicol nanogels group. Physiological saline, N, O-CMCS, OHA, blank nanogels, and florfenicol nanogels (100 μL each) were applied to the wounds in the control, N, O-CMCS, OHA, blank nanogels, and florfenicol nanogels groups, respectively. The trial came to an end when the wounds in the florfenicol nanogels group healed completely. Up to the endpoint, days 0, 2, 5, 9, 13, and 15 were used to take pictures of the wounds in each group. The size of the wound area was examined using Image J analysis software 1.8.0 (National Institutes of Health, Bethesda, MD, USA). The original wound percentage (%) = (S_o_ − S_t_ /S_o_) × 100% was used to determine the wound healing rate, where S_o_ denotes the size of the wound at the beginning and S_t_ denotes the size of the wound at the time of measurement. Furthermore, on days 0, 2, 5, 9, 13, and 15, the skin tissues of the wound sites were collected for staining with hematoxylin and eosin (H&E).

### 4.11. In Vivo Antibacterial Studies

These investigations used infected wounds from mouse models to evaluate the in vivo antibacterial activity of N, O-CMCS, OHA, blank nanogels, and florfenicol nanogels. In summary, on days 0, 2, 5, 9, 13, and 15, wound exudates were collected using sterile swabs from several groups (control, N, O-CMCS, OHA, blank nanogels, and florfenicol nanogels). The samples were diluted 10,000 times and then spread out on agar plates after being amplified in liquid beef culture media for 4 h at 37 °C. Following an 18 h incubation period at 37 °C, the colony counts were determined. Bacterial inhibition percent, or (C_con_ − C_exp_)/C_con_ × 100%, was used to quantify the antibacterial activity of various treatments. C_con_ and C_exp_ stand for CFUs in the control and experimental (N, O-CMCS, OHA, blank nanogels, and florfenicol nanogels) groups, respectively.

### 4.12. Statistical Analysis

The experimental data are expressed as mean ± S.D. and analyzed by a one-way ANOVA using the SPSS 19.0 software. The statistical significance was determined using (*p*-value < 0.05).

## Data Availability

The original contributions presented in this study are included in the article/Supplementary Materials. Further inquiries can be directed to the corresponding authors.

## Supplementary Materials

The following supporting information can be downloaded at: https://www.mdpi.com/article/10.3390/gels11060380/s1, Figure S1: PXRD of CS and N, O-CMCS. Figure S2: FTIR spectrum of CS and N, O-CMCS. Figure S3: ^1^H-NMR and ^1^C-NMR spectrum of CS. Figure S4: ^1^H-NMR and ^1^C-NMR spectrum of N, O-CMCS. Figure S5: Appearance of CS and N, O-CMCS. Figure S6: Freeze-dried samples of CS and N, O-CMCS. Figure S7: Resolvability of CS and N, O-CMCS. Figure S8: SEM of N, O-CMCS. Figure S9: TEM of N, O-CMCS. Figure S10: EDS of freeze-dried N, O-CMCS. Figure S11: ZP of freeze-dried N, O-CMCS. Figure S12: PXRD of HA and OHA. Figure S13: FTIR spectrum of HA and OHA. Figure S14: ^1^H-NMR and ^1^C-NMR spectrum of HA. Figure S15: ^1^H-NMR and ^1^C-NMR spectrum of OHA. Figure S16: Appearance of HA and OHA. Figure S17: Freeze-dried samples of HA and OHA. Figure S18: Resolvability of HA and OHA. Figure S19: SEM of OHA. Figure S20: TEM of OHA. Figure S21: EDS of freeze-dried OHA. Figure S22: ZP of HA and OHA. Figure S23: Standard curve of florfenicol. Figure S24: Injectability of florfenicol nanogels. Figure S25: Changes in glucose. Figure S26: Quantitative analysis of ROS in *E. coli* (A) and *S. aureus* (B) in different treatment groups. Table S1: Single-factor experimental design and values of the responses for the florfenicol nanogels. Table S2: ANOVA of the LC model. Table S3: ANOVA of EE model. Table S4: The influence factors test of florfenicol nanogels. Table S5: Primer sequences for RT-qPCR.
