# Supplementary material for: Nanogel Dressing with Targeted Glucose Reduction and pH/Hyaluronidase Dual-Responsive Release for Synergetic Therapy of Diabetic Bacterial Wounds
Source: Gels. 2025 May 22;11(6):380. doi: 10.3390/gels11060380 (PMC12192132; doi:10.3390/gels11060380)
Supplement: Supplementary file 1 [file gels-11-00380-s001.zip › gels-3604214-supplementary.pdf]

Supplementary Materials for

Nanogel Dressing with Targeted Glucose Reduction and pH/Hyaluronidase Dual-Responsive Release for Synergetic Therapy of Diabetic Bacterial Wounds

Wanhe Luo [1,*,†], Yongtao Jiang [1,†], Jinhuan Liu [1,2,†], Samah Attia Algharib [3], Ali Sobhy Dawood [4] and Shuyu Xie [5,*]

[1] Engineering Laboratory for Tarim Animal Diseases Diagnosis and Control, College of Animal Science and Technology, Tarim University, Alar 843300, China; jiangyongtao2022@163.com (Y.J.); liujinhuan0830@163.com (J.L.)
[2] College of Veterinary Medicine, Sichuan Agricultural University, Chengdu 611130, China
[3] Department of Clinical Pathology, Faculty of Veterinary Medicine, Benha University, Moshtohor 13736, Egypt; samah.alghareeb@fvtm.bu.edu.eg
[4] Infectious Diseases, Faculty of Veterinary Medicine, University of Sadat City, Sadat City 32897, Egypt; ali.dawood@vet.usc.edu.eg
[5] National Reference Laboratory of Veterinary Drug Residues (HZAU), Huazhong Agricultural University, Wuhan 430070, China
* Correspondence: 120200015@taru.edu.cn (W.L.); xieshuyu@mail.hzau.edu.cn (S.X.)
† These authors contributed equally to this work.

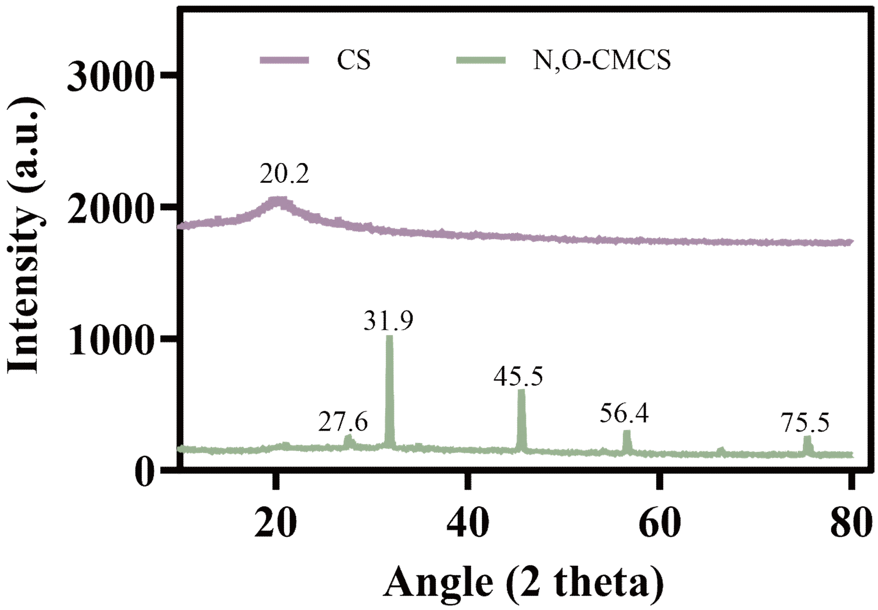

Figure S1. PXRD of CS and N,O-CMCS.

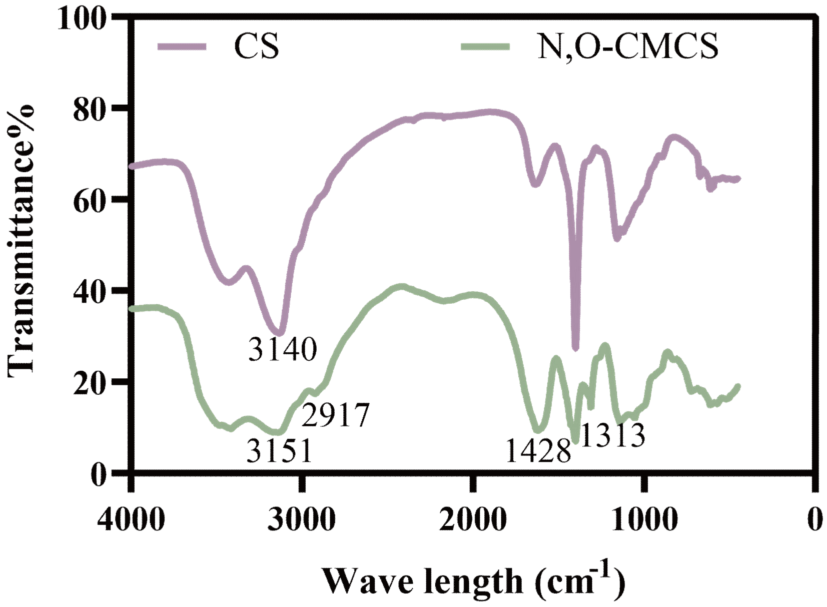

Figure S2. FTIR spectrum of CS and N,O-CMCS.

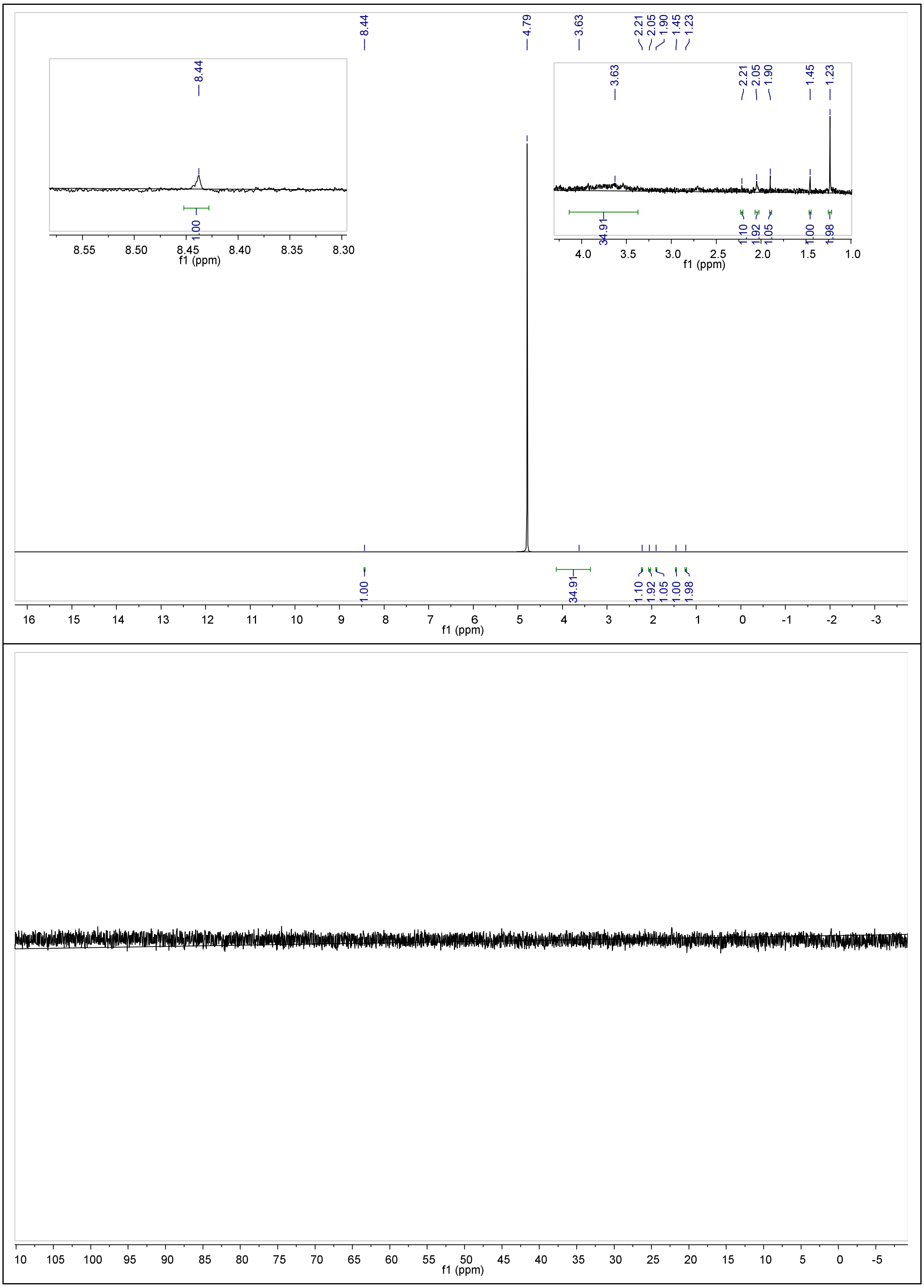

Figure S3. ^{1}H-NMR and ^{1}C-NMR spectrum of CS.

^{1}H NMR (400 MHz, D_2O) δ 8.44 (s, 1H), 3.63 (s, 35H), 2.21 (s, 1H), 2.05 (s, 2H), 1.90 (s, 1H), 1.45 (s, 1H), 1.23 (s, 2H).

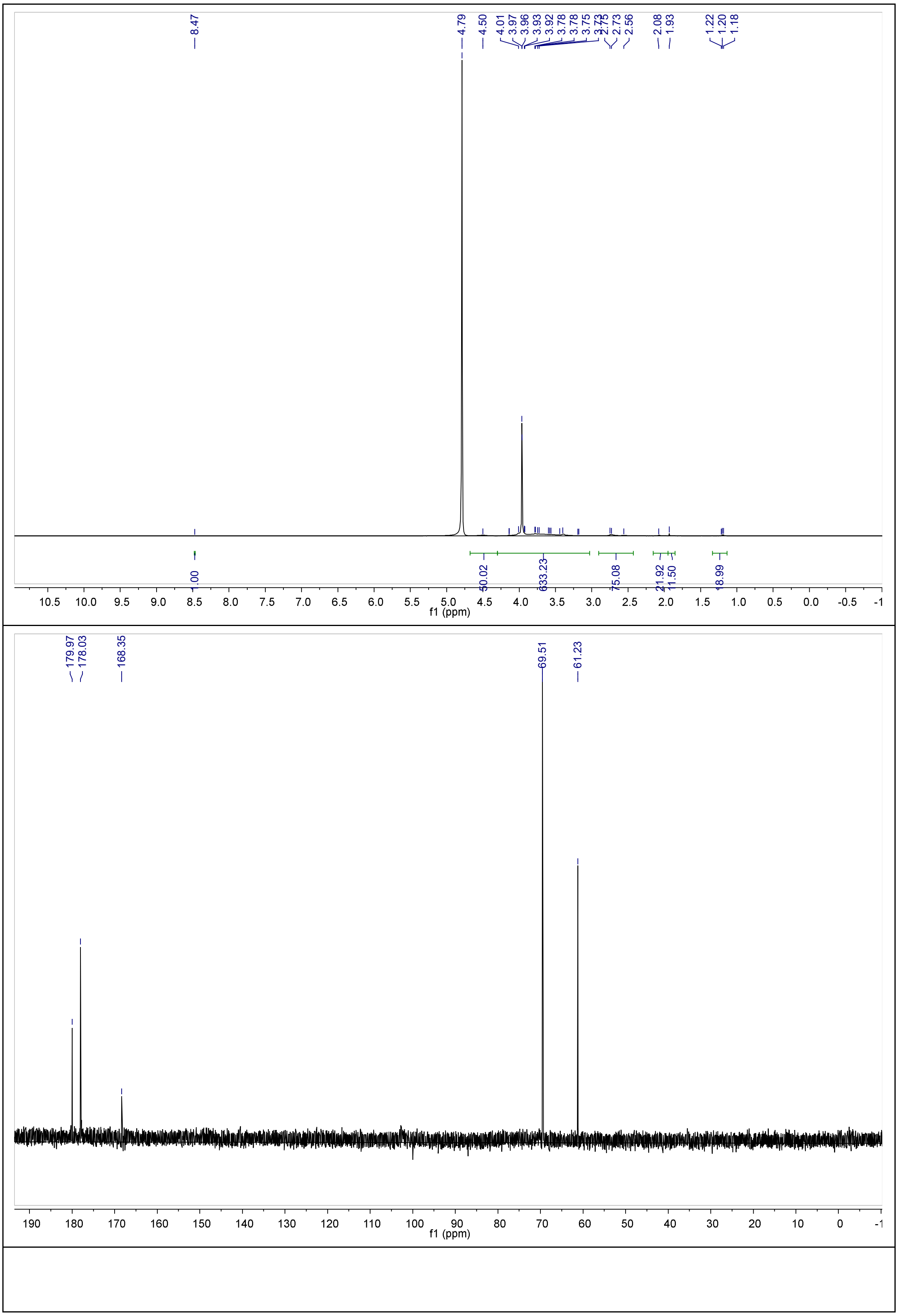

Figure S4. ^{1}H-NMR and [1]C-NMR spectrum of N,O-CMCS.

^{1}H NMR (400 MHz, D_2O) δ 8.47 (s, 1H), 4.50 (s, 50H), 4.30 – 3.03 (m, 633H), 2.90 – 2.43 (m, 75H), 2.08 (s, 22H), 1.93 (s, 11H), 1.34 – 1.14 (m, 19H).

^{13}C NMR (101 MHz, D_2O) δ 179.97, 178.03, 168.35, 69.51, 61.23.

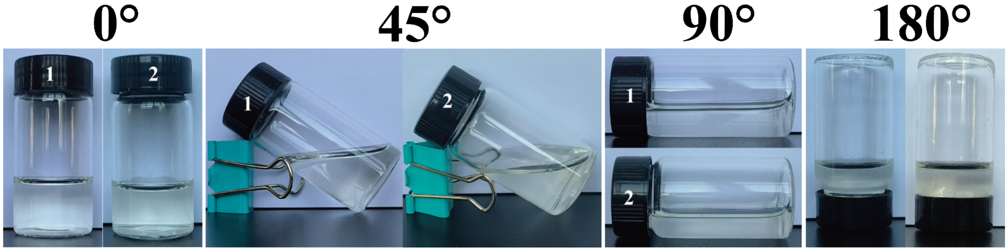

Figure S5. Appearance of CS (1) and N,O-CMCS (2).

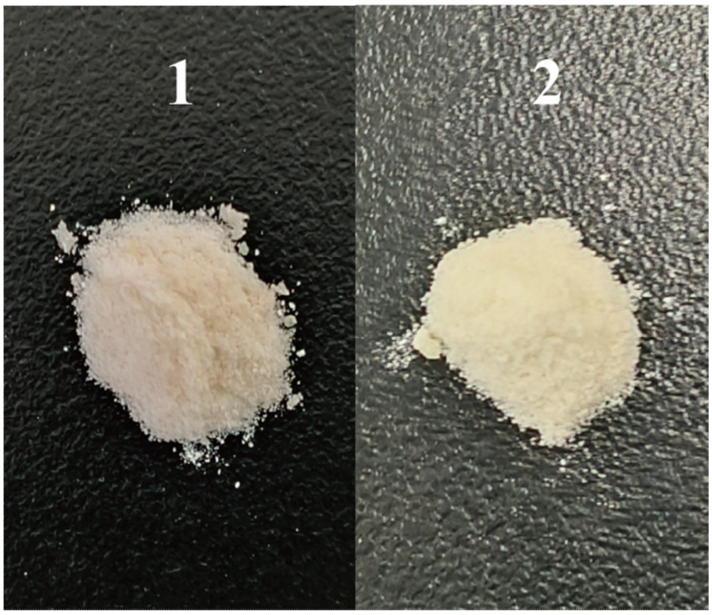

Figure S6. Freeze-dried samples of CS (1) and N,O-CMCS (2).

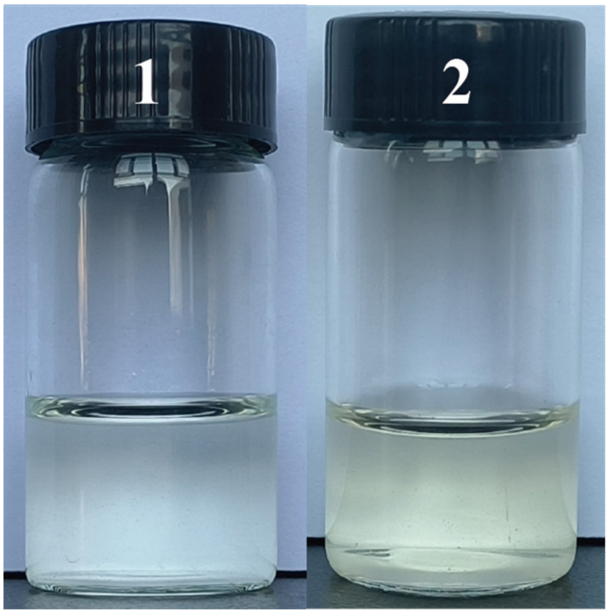

Figure S7. Resolvability of CS (1) and N,O-CMCS (2).

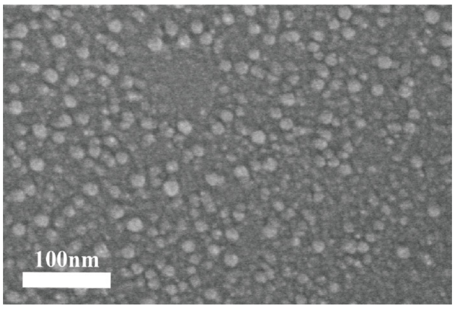

Figure S8. SEM of N,O-CMCS.

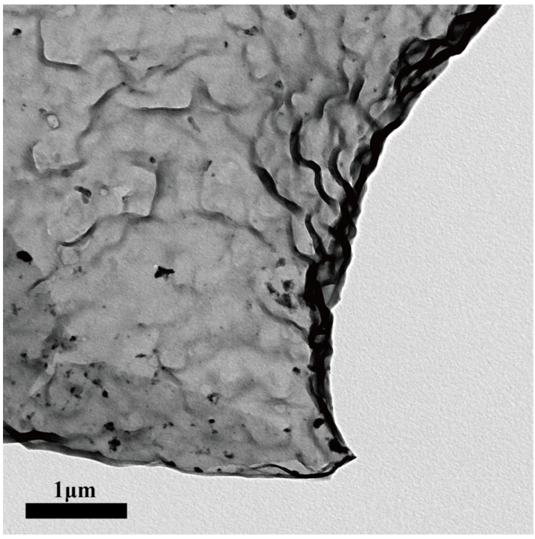

Figure S9. TEM of N,O-CMCS.

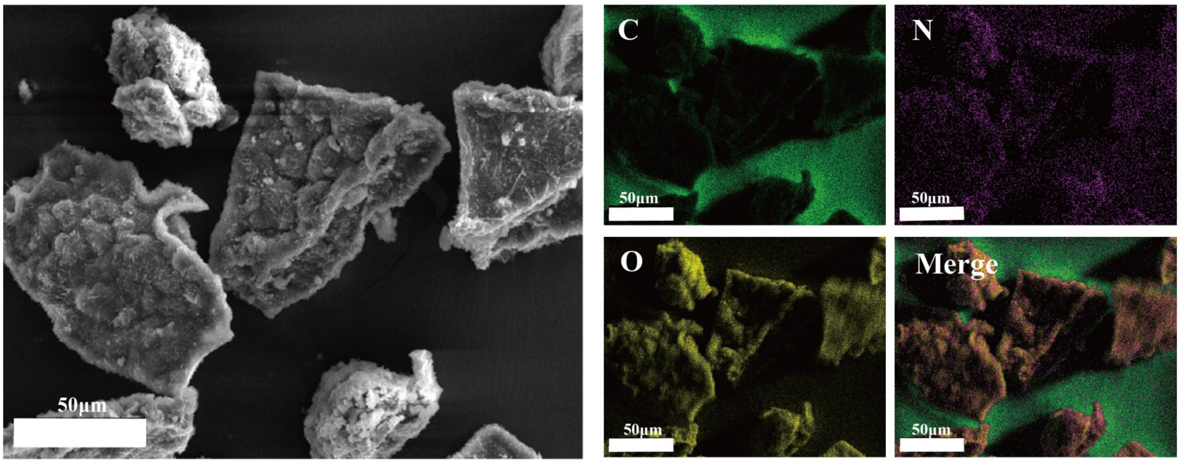

Figure S10. EDS of freeze-dried N,O-CMCS.

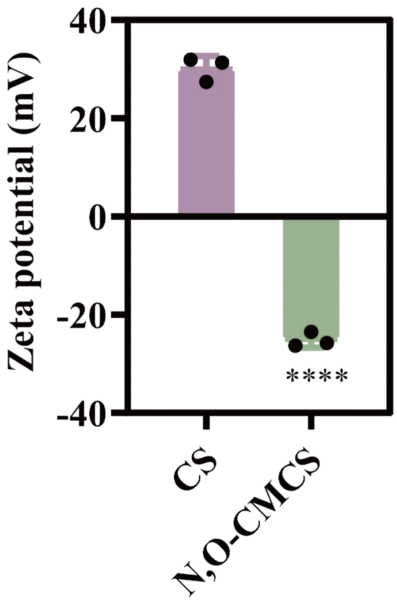

Figure S11. ZP of freeze-dried N,O-CMCS.

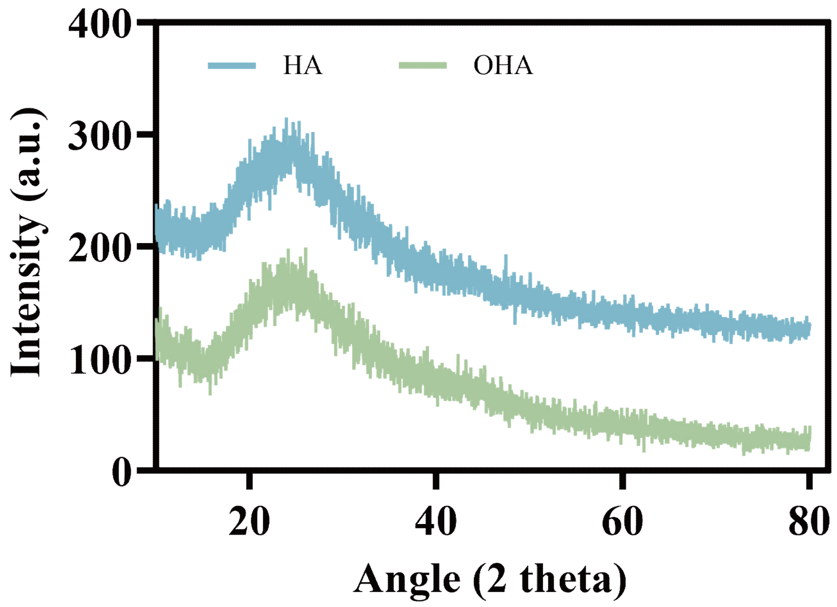

Figure S12. PXRD of HA and OHA.

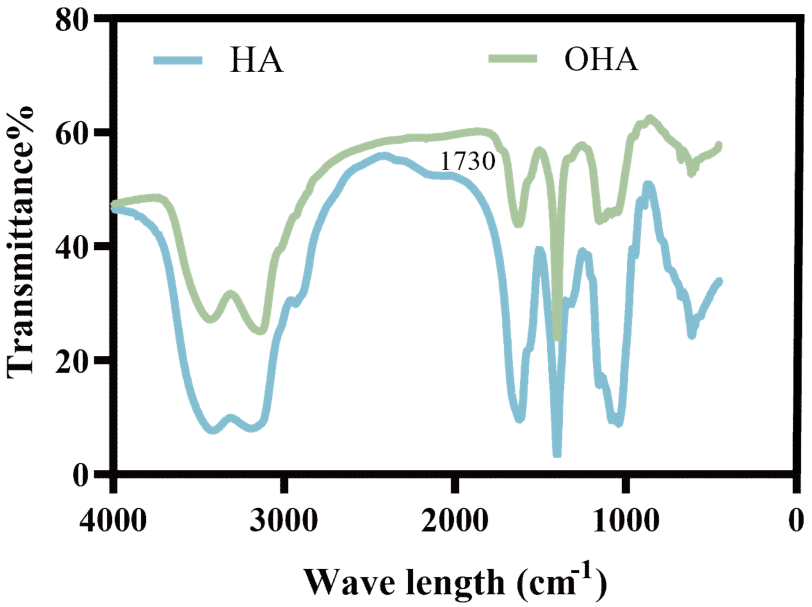

Figure S13. FTIR spectrum of HA and OHA.

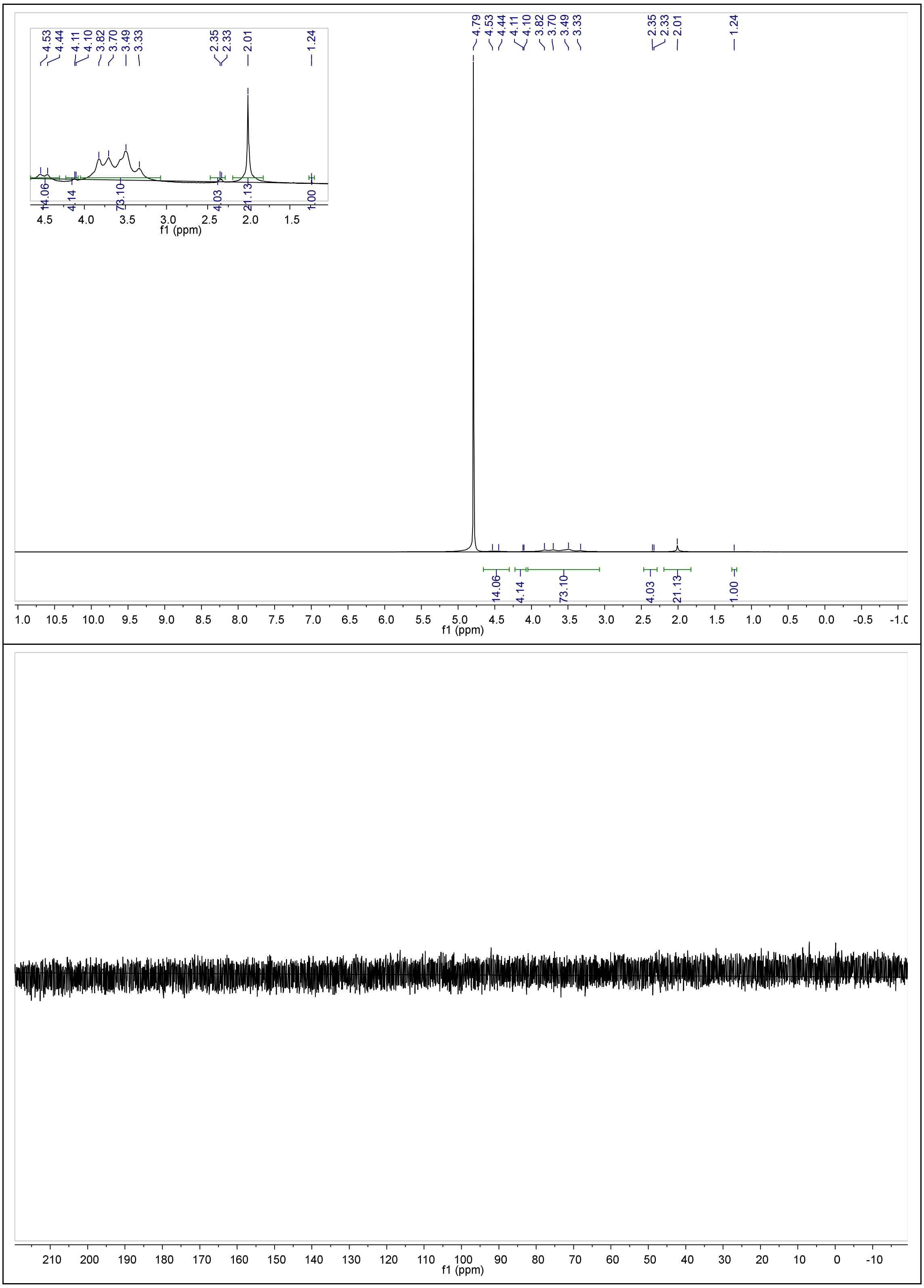

Figure S14. ^{1}H-NMR and ^{1}C-NMR spectrum of HA.

^{1}H NMR (400 MHz, D_2O) δ 4.49 (d, J = 34.4 Hz, 14H), 4.11 (d, J = 5.7 Hz, 4H), 3.59 (dd, J = 140.2, 56.6 Hz, 73H), 2.34 (d, J = 8.1 Hz, 4H), 2.01 (s, 21H), 1.24 (s, 1H).

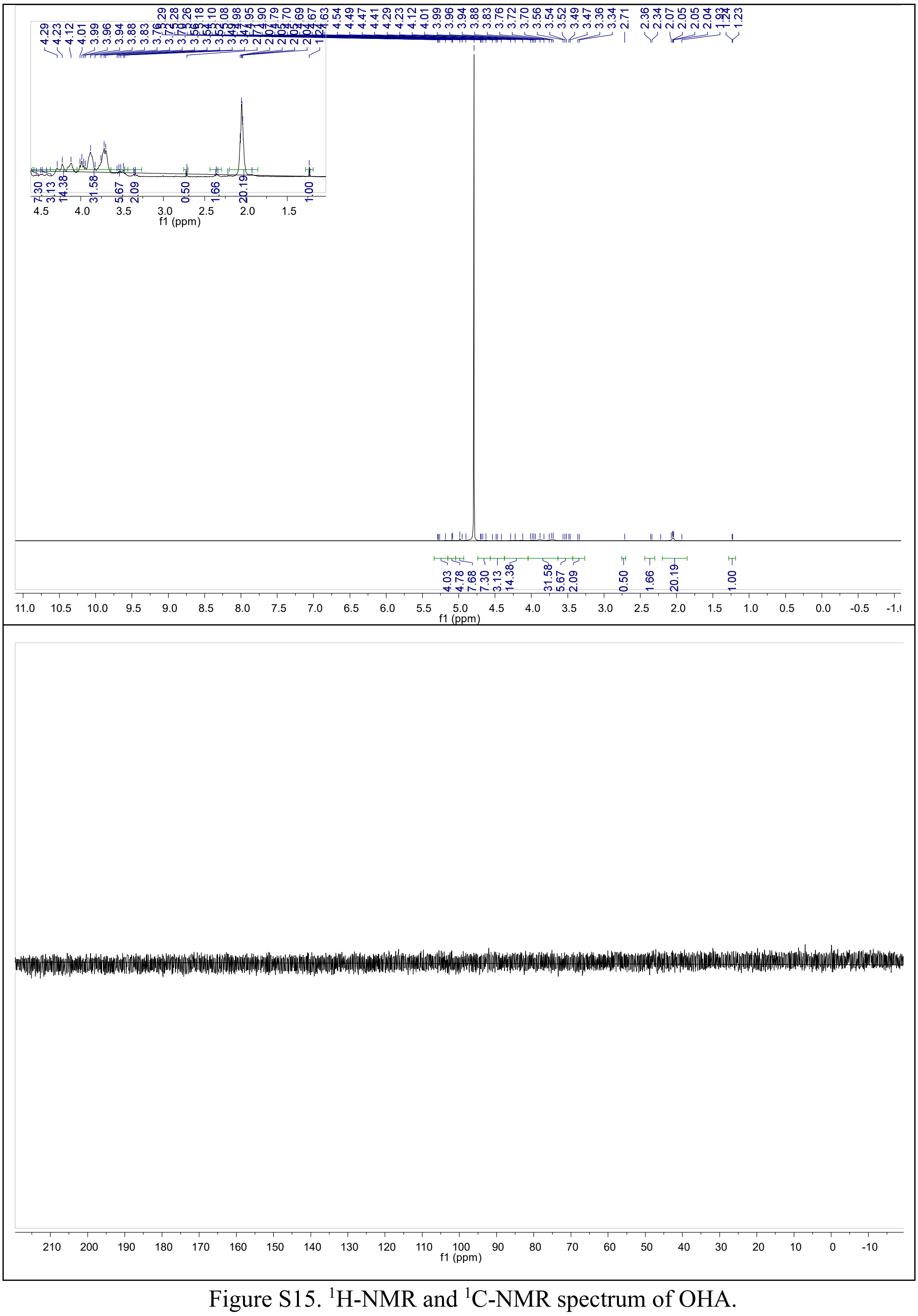

Figure S15. ^{1}H-NMR and [1]C-NMR spectrum of OHA.

^{1}H NMR (400 MHz, D_2O) δ 5.34 – 5.15 (m, 4H), 5.09 (d, J = 4.5 Hz, 5H), 4.97 (d, J = 12.4 Hz, 8H), 4.67 (dd, J = 19.2, 10.9 Hz, 7H), 4.48 (dd, J = 29.1, 21.5 Hz, 3H), 4.37 – 4.04 (m, 14H), 4.04 – 3.63 (m, 32H), 3.63 – 3.43 (m, 6H), 3.35 (d, J = 8.3 Hz, 2H), 2.71 (s, 1H), 2.35 (d, J = 7.6 Hz, 2H), 2.20 – 1.86 (m, 20H), 1.23 (d, J = 4.1 Hz, 1H).

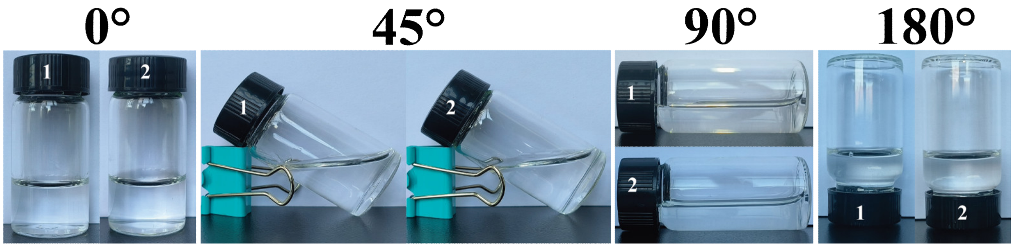

Figure S16. Appearance of HA (1) and OHA (2).

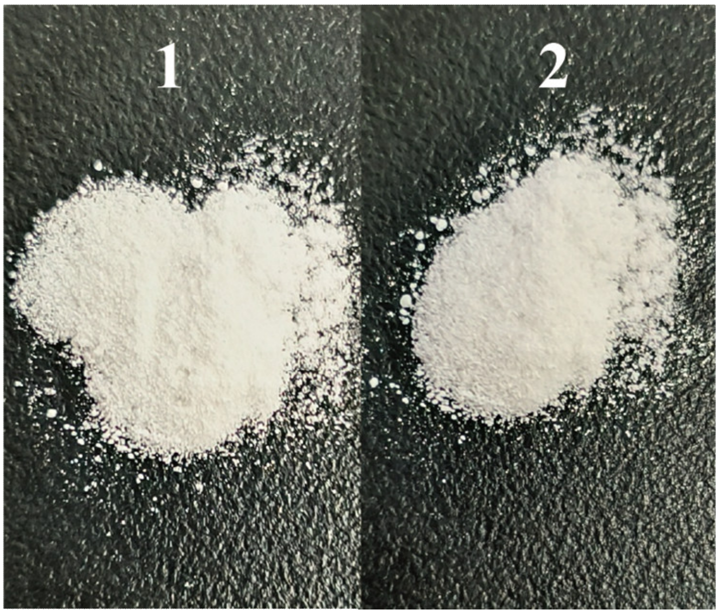

Figure S17. Freeze-dried samples of HA (1) and OHA (2).

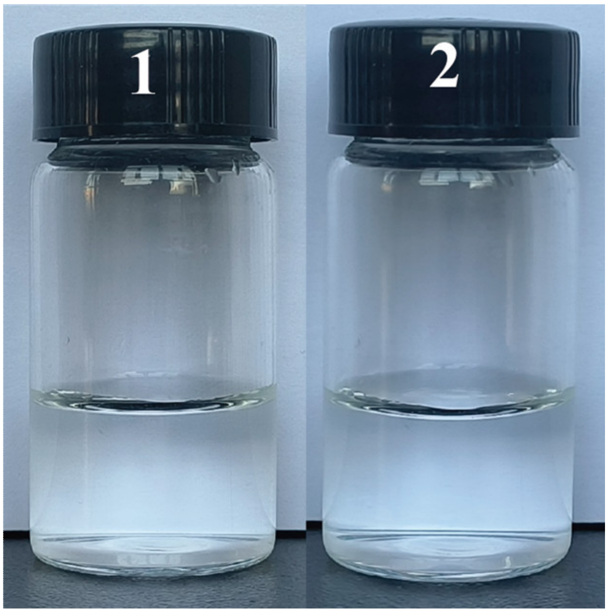

Figure S18. Resolvability of HA (1) and OHA (2).

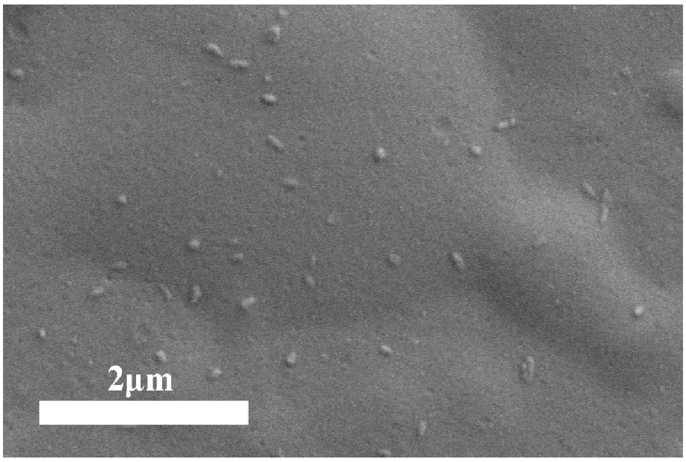

Figure S19. SEM of OHA.

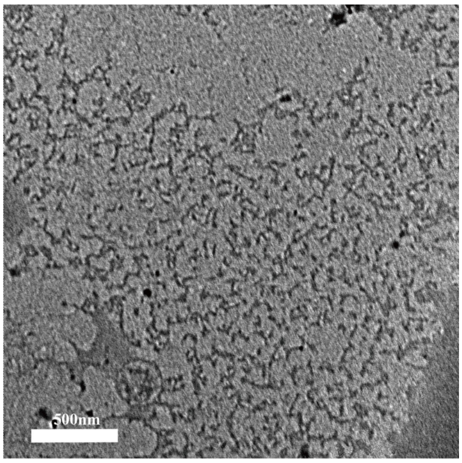

Figure S20. TEM of OHA.

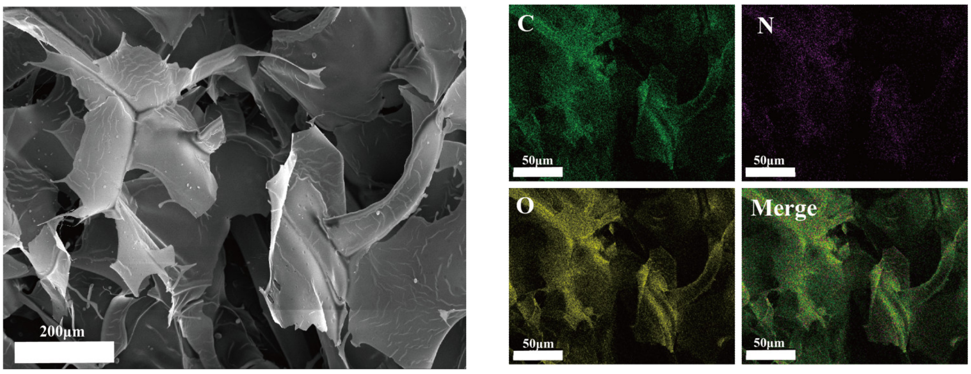

Figure S21. EDS of freeze-dried OHA.

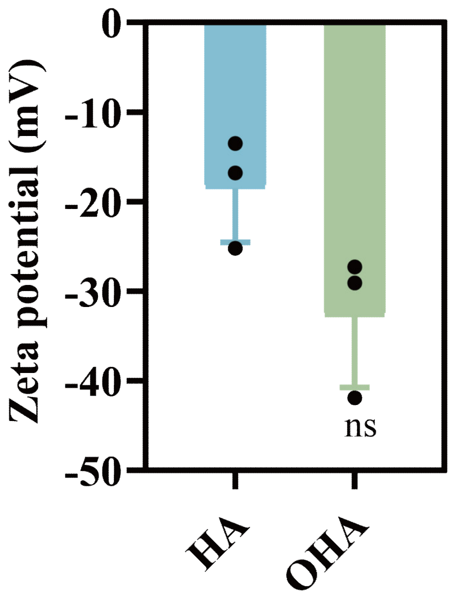

Figure S22. ZP of HA and OHA.

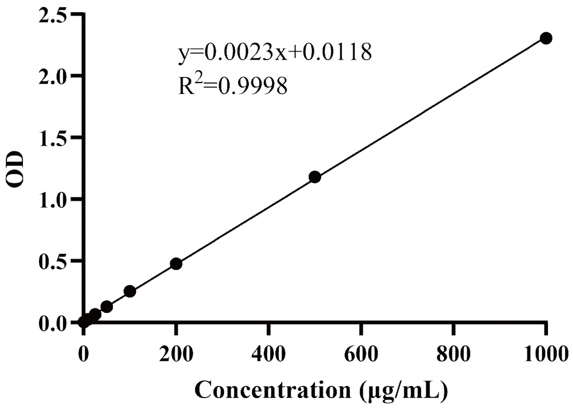

Figure S23. Standard curve of florfenicol.

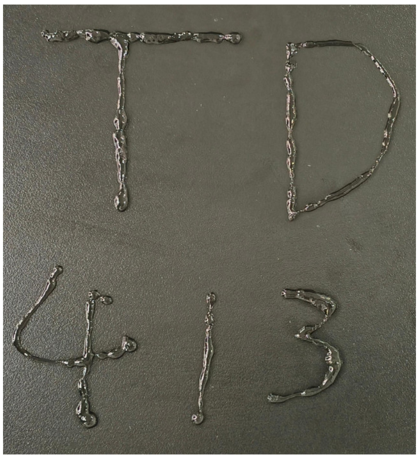

Figure S24. Injectability of florfenicol nanogels.

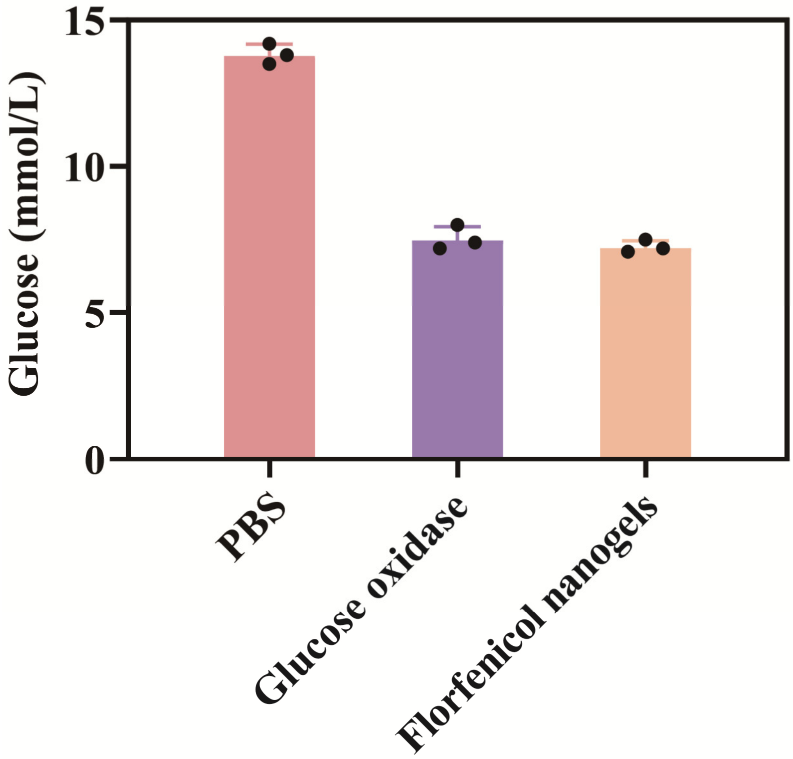

Figure S25. Changes in glucose.

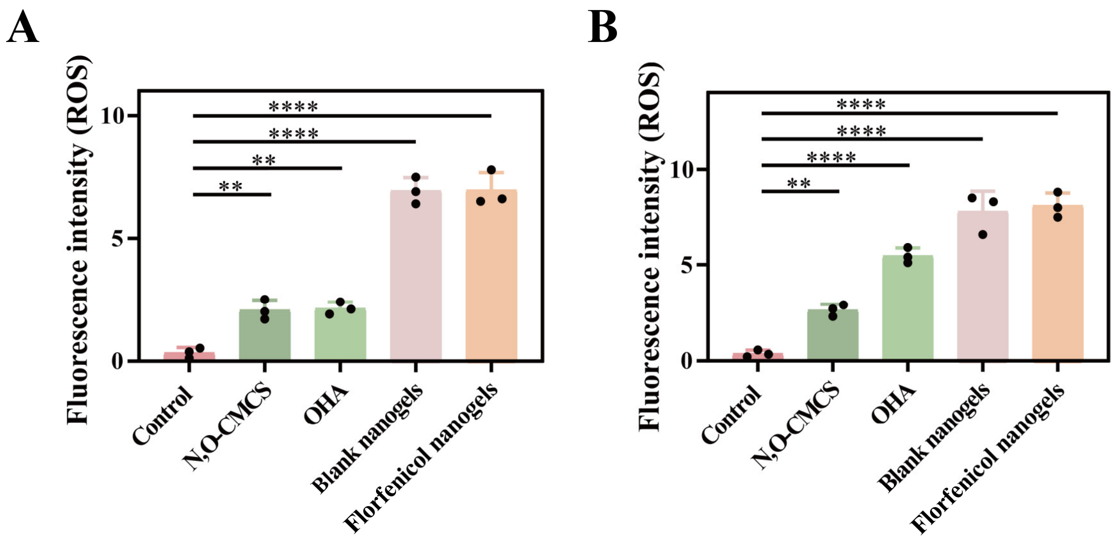

Figure S26. Quantitative analysis of ROS in *E. coli* (A) and *S. aureus* (B) in different treatment groups.

Table S1. Single-factor experimental design and values of the responses for the florfenicol nanogels.

	N,O-CMCS (mg)	OHA (mg)	Loading capacity (%)	Encapsulation efficiency (%)
1	150	12.5	54.1	75.8
2	12.5	12.5	54.5	73.0
3	50	50	62.4	84.3
4	50	50	62.4	84.3
5	50	50	62.4	84.3
6	50	50	62.4	84.3
7	150	150	60.3	80.5
8	150	50	61.8	81.9
9	12.5	50	58.5	79.0
10	12.5	150	54.5	76.7
11	50	12.5	57.5	78.5
12	50	150	61.3	82.8
13	50	50	62.4	84.3

Table S2. ANOVA of LC model.

Source	Sum of Squares	df	Mean Square	F-value	P-value
Model	133.85	5	26.77	129.34	<0.0001
A-N,O-CMCS	12.61	1	12.61	60.95	0.0001
B-OHA	16.67	1	16.67	80.52	<0.0001
AB	9.61	1	9.61	46.43	0.0003
A^2	22.87	1	22.87	110.49	<0.0001
B^2	36.34	1	36.34	175.60	<0.0001
Residual	1.45	7	0.2070		
Lack of Fit	1.45	3	0.4830		
Pure Error	0.0000	4	0.0000		
Cor Total	135.30	12			
R^2	0.9893				
Adj-R^2	0.9816				
Pre-R^2	0.9200				
Adeq precision	26.7630				
CV%	0.7636				

Table S3. ANOVA of EE model.

Source	Sum of Squares	df	Mean Square	F-value	P-value
Model	177.57	5	35.51	2093.75	<0.0001
A-N,O-CMCS	15.04	1	15.04	886.77	<0.0001
B-OHA	26.88	1	26.08	1584.80	<0.0001
AB	0.2500	1	0.2500	14.74	0.0064
A^2	44.08	1	44.08	2598.49	<0.0001
B^2	39.77	1	39.77	2344.82	<0.0001
Residual	0.1187	7	0.0170		
Lack of Fit	0.1187	3	0.0396		
Pure Error	0.0000	4	0.0000		
Cor Total	177.69	12			
R^2	0.9993				
Adj-R^2	0.9989				
Pre-R^2	0.9944				
Adeq precision	127.0304				
CV%	0.1613				

Table S4. The influence factors test of florfenicol nanogels (Mean±SD, n = 3).

	5 d			10 d		
Influencing factor	Size	ZP	PDI	Size	ZP	PDI
High temperature	325.1±1.0	-29.1±0.6	0.15±0.08	321.0±2.1	-29.1±0.6	0.30±0.04
High humidity	331.5±1.5	-28.5±0.8	0.22±0.15	326.5±2.2	-28.8±0.7	0.30±0.11
Strong light	324.3±2.7	-28.7±1.5	0.21±0.07	325.3±1.3	-29.7±0.6	0.17±0.15

Table S5. Primer sequences for RT-qPCR.

Gene name	Sense Primer
agrA-F	GCCCTCGCAACTGATAATCC
agrA-R	GGTCATGCTTACGAATTTCACTG
agrC-F	AAATTGATGACCCTATCATTCGC
agrC-R	ATAGACCTAAACCACGACCTTC
icaA-F	TGAACCGCTTGCCATGTG
icaA-R	CACGCGTTGCTTCCAAAGA
icaR-F	TCGAACTATTCAATTGATGCTTTA
icaR-R	CAGAAAATTCCTCAGGCGTA
